# Characterization of a *Dmd*^*EGFP*^ reporter mouse as a tool to investigate dystrophin expression

**DOI:** 10.1186/s13395-016-0095-5

**Published:** 2016-07-05

**Authors:** Mina V. Petkova, Susanne Morales-Gonzales, Karima Relizani, Esther Gill, Franziska Seifert, Josefine Radke, Werner Stenzel, Luis Garcia, Helge Amthor, Markus Schuelke

**Affiliations:** Department of Neuropediatrics, Charité—Universitätsmedizin Berlin, Augustenburger Platz 1, 13353 Berlin, Germany; NeuroCure Clinical Research Center, Charité—Universitätsmedizin Berlin, Berlin, Germany; Université de Versailles St-Quentin, INSERM U1179 and LIA BAHN Centre Scientifique de Monaco, Montigny-le Bretonneux, France; Institute of Neuropathology, Charité—Universitätsmedizin Berlin, Berlin, Germany

**Keywords:** Dystrophin, Dmd, EGFP, Duchenne muscular dystrophy, Reporter mouse

## Abstract

**Background:**

Dystrophin is a rod-shaped cytoplasmic protein that provides sarcolemmal stability as a structural link between the cytoskeleton and the extracellular matrix via the dystrophin-associated protein complex (DAPC). Mutations in the dystrophin-encoding *DMD* gene cause X-linked dystrophinopathies with variable phenotypes, the most severe being Duchenne muscular dystrophy (DMD) characterized by progressive muscle wasting and fibrosis. However, dystrophin deficiency does not only impair the function of skeletal and heart muscle but may also affect other organ systems such as the brain, eye, and gastrointestinal tract. The generation of a dystrophin reporter mouse would facilitate research into dystrophin muscular and extramuscular pathophysiology without the need for immunostaining.

**Results:**

We generated a *Dmd*^*EGFP*^ reporter mouse through the in-frame insertion of the *EGFP *coding sequence behind the last *Dmd *exon 79, which is known to be expressed in all major dystrophin isoforms. We analyzed EGFP and dystrophin expression in various tissues and at the single muscle fiber level. Immunostaining of various members of the DAPC was done to confirm the correct subsarcolemmal location of dystrophin-binding partners. We found strong natural EGFP fluorescence at all expected sites of dystrophin expression in the skeletal and smooth muscle, heart, brain, and retina. EGFP fluorescence exactly colocalized with dystrophin immunostaining. In the skeletal muscle, dystrophin and other proteins of the DAPC were expressed at their correct sarcolemmal/subsarcolemmal localization. Skeletal muscle maintained normal tissue architecture, suggesting the correct function of the dystrophin-EGFP fusion protein. EGFP expression could be easily verified in isolated myofibers as well as in satellite cell-derived myotubes.

**Conclusions:**

The novel dystrophin reporter mouse provides a valuable tool for direct visualization of dystrophin expression and will allow the study of dystrophin expression in vivo and in vitro in various tissues by live cell imaging.

**Electronic supplementary material:**

The online version of this article (doi:10.1186/s13395-016-0095-5) contains supplementary material, which is available to authorized users.

## Background

Duchenne muscular dystrophy (DMD; OMIM#310200), the most severe form of dystrophinopathies, is a lethal X-linked recessive disorder characterized by progressive muscle degeneration, loss of walking ability, decline of respiratory and cardiac function, and early death [1]. Besides the skeletal and heart muscle [2], the disease affects other organs such as the central nervous system [3] and organs which are dependent on smooth muscle function such as the gastrointestinal [4] and the vascular system [5]. DMD is caused by mutations in the dystrophin gene (*DMD*) that prevent the expression of functional dystrophin. The dystrophin gene is the largest human gene spanning 2.4 Mb at the Xp21 locus and comprises 79 primary exons [6–8].

The main dystrophin isoform has a molecular weight of 427 kDa and is expressed in muscle cells, where it is localized at the cytoplasmatic side of the sarcolemma [9, 10]. Dystrophin has four main functional domains: (i) the N-terminal actin-binding domain, (ii) the central rod domain, (iii) the cysteine-rich, and (iv) the C-terminal domain. The N-terminus and part of the dystrophin rod domain interact with cytoskeletal actin [11, 12]. The rod domain folds into α-helical coils composed of 24 spectrin-like repeats interrupted by four proline-rich hinges that lend flexibility to the protein [13, 14]. Dystrophin assembles several transmembrane and cytoplasmic proteins into the dystrophin-associated protein complex (DAPC), interactions provided by the cysteine-rich and C-terminal domains. The DAPC can be divided into (i) the dystroglycan complex, (ii) the sarcoglycan complex, and (iii) the cytoplasmic and extracellular matrix (ECM) components including sarcospan, α-laminin, syntrophins (β, γ2), dystrobrevin, and neuronal nitric oxide synthase (nNOS) [15, 16]. The DAPC connects the cytoskeleton with the ECM, thereby lending mechanical stability to the sarcolemma and protecting the muscle from contraction-related muscle damage [9, 10]. The importance of dystrophin in DAPC assembly is highlighted by the loss of DAPC components in DMD muscle.

Vast progress has been made in the last two decades to comprehend the role of dystrophin in the muscle. Prominent contributions to the advancement of this field have been obtained through identification and later also the generation of animal models (e.g., *mdx* mice, GRMD dogs) [17, 18].

However, despite the insight into the pathophysiology of dystrophinopathies, DMD cannot be cured at present and the extramuscular functions of dystrophin are not well understood. This calls for further investigations into the (patho)physiology of dystrophin, not only in the muscle but also in other organs and tissues where it is expressed. The character of DMD as a multisystem disorder is reflected by a large number of highly conserved isoforms and splicing variants that differ in their cellular and subcellular localization. The high diversity of dystrophin isoforms is achieved through the use of tissue-specific promoters (Additional file 1: Figure S1). The full-length dystrophin isoform, Dp427, is generated from three different tissue-specific promoters: (i) muscle type (M), which drives the expression of skeletal, cardiac, and smooth muscle dystrophin, (ii) brain type (B), which is active in cortical and cerebellar neurons and heart, and (iii) Purkinje cell type (P), which regulates the cerebellar dystrophin expression [19–22]. Shorter dystrophin isoforms such as the retinal (Dp260), the cerebellar and renal (Dp140) [23], as well as the Schwann cell (Dp116) isoforms [24] are transcribed from internal promoters. The Dp260, Dp116, and Dp140 isoforms include parts of the rod domain and express the cysteine-rich and C-terminal domains, but lack the N-terminal actin-binding domain [23–25]. The short Dp71 isoform is detected in most non-muscle tissues including the brain, liver, kidney, and lung [26–29]. Further dystrophin diversification is achieved by alternative splicing throughout the coding sequence of dystrophin [20, 30]. Notably, two alternative splicing sites exist at the 3′-end of the *DMD* gene [31]. Their usage has been well characterized in the Dp71 isoform. In the Dp71d isoform, excision of exon 71 does not alter the reading frame of the transcript and still generates the 13-amino-acid-long C-terminus common to most dystrophin isoforms including exon 79. The Dp71f (Dp71b) isoform, however, is generated by alternative splicing of exon 78 which shifts the reading frame and produces a C-terminus that contains 31 new amino acids with hydrophobic properties (Additional file 1: Figure S1). Another isoform is Dp71c lacking exon 71-74 that encode the 110-amino-acid sequence of the syntrophin-binding domain. Moreover, exon 78 can be additionally skipped in the latter isoform creating the Dp71_Δ110_ variant. The expression of these isoforms is differentially regulated during human embryonic development and adulthood [20, 27, 31, 32]. Similar expression patterns have been observed in animal models on the mRNA and protein level [20, 33]. The shortest dystrophin isoform, Dp40, has the same promoter as Dp71 but lacks the normal C-terminal end of Dp427. Although less abundant Dp40 shows a similar expression pattern as Dp71 [34].

In the nervous system, all the different dystrophin isoforms have been identified. They are expressed not only in the adult but also during neural development. DMD patients may suffer from CNS dysfunction including cognitive [15, 35] and visual impairment [25, 36–38]. However, the exact role of dystrophin in the CNS as well as its contribution to the CNS phenotype of DMD patients is still a matter of debate and hampered by the complexity and high variety of the dystrophin isoforms and their DAPC components: Dp427 is expressed at the postsynapse of neurons in the *hippocampus*, *cerebellum*, and cerebral cortex [39–43]. An involvement of this isoform in neuronal activity as well as in the formation and maintenance of the blood-brain barrier has been suggested [44–47]. The Dp140 isoform is mainly expressed during development [23]. Dp71 is expressed in glial cells, notably in perivascular astrocytes and the Bergmann glia of the *cerebellum* and in the Müller glia of the retina that surrounds the endothelial cells [23, 48]. In the brain and retina, several authors proposed a role of Dp71 in the maintenance of potassium and water homeostasis as well as in the regulation of vascular permeability [49–53]. In the retina, Dp427 and Dp260 isoforms are associated with photoreceptor terminals suggesting the involvement of dystrophin in synaptic transmission, but the precise mechanism is still unclear [38, 54, 55].

Overall, there are still many questions to be answered about the role of dystrophin in health and disease, especially in non-muscle tissues. Interestingly, the recent discovery of a role for dystrophin in satellite cells points to the fact that there are still unknown functions to be found that might open new leads in DMD research [56]. Therefore, it would be beneficial to have a model organism to facilitate the study of dystrophin expression from its natural promoter(s) in various tissues without the need for immunostaining. Hence, we set out to generate a *Dmd*^*EGFP*^ reporter mouse, tagging the C-terminus of the protein with EGFP and verifying its proper subcellular expression in various mouse organs.

## Methods

### Generation of transgenic mice

**Fig. 1:**
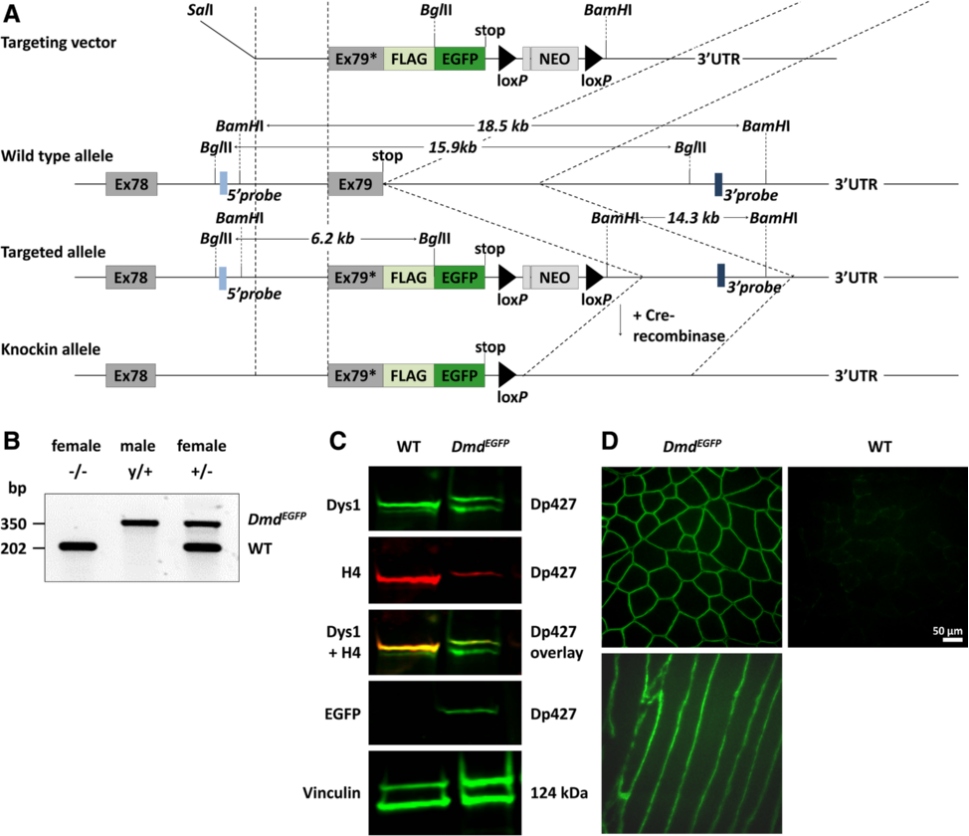
Generation of the *Dmd* ^*EGFP*^ reporter mouse. **a** Construction of the targeting vector through insertion of a FLAG-EGFP coding sequence into the 3′UTR of the *Dmd* gene. The termination codon of exon 79 was altered to a leucine coding triplet. The *asterisk* indicates the modified exon 79. The *Dmd* wild-type allele was targeted in ES cells after electroporation of the *Sal*I linearized targeting vector and subsequent homologous recombination. *Dashed lines* represent homologous regions. *Bgl*II and *Bam*HI restriction sites were artificially inserted into the construct to facilitate identification of targeted ES cells by Southern blot analysis using either 5′ and 3′ hybridization probes (*blue squares*) located outside of the targeting vector. Recombinant ES cells were then used for generation of transgenic mice. The *neo* cassette was removed by crossing the F1 generation mice with ubiquitous Cre-deleter mice (constructs are not drawn to scale). **b** PCR genotyping of the wild-type (202 bp) and recombinant (350 bp) allele. *−/−* homozygous wild-type female, *y/+* hemizygous male for the *Dmd* ^*EGFP*^ allele, *+/−* heterozygous female. **c** Western blot analysis of whole TA muscle extract using dystrophin antibodies against the rod (Dys1) and the C-terminal domain (H4) with normal expression of the full-length Dp427 dystrophin in the *Dmd* ^*EGFP*^ mice. The anti-GFP antibody detected the tagged Dp427-EGFP protein only in the transgenic animal. **d** TA cross and longitudinal sections show natural EGFP fluorescence (*green*) in *Dmd* ^*EGFP*^ mice that can be detected at the *sarcolemma* without amplification of the signal. No fluorescent signal above background was observed in the wild-type cross section using the identical illumination parameters

A targeting vector was generated in order to modify the dystrophin gene (*Dmd*) by inserting a FLAG and a modified EGFP-L221K sequence [57] downstream of the last exon 79 using the recombineering and gap repair technique [58–60] (Fig. 1a). Briefly, a 10.943-kb DNA fragment spanning intron 78, exon 79, and part of the 3′UTR of murine *Dmd* was amplified from a genomic X-chromosomal BAC clone (bMQ-389G17, Source Bioscience Nottingham, UK) and subcloned into the pCR-Script vector (Addgene, Cambridge, MA, USA). *Dmd* exon 79 was then modified by changing the stop codon into a leucine and inserting a FLAG-EGFP sequence in-frame, followed by a neomycin cassette (*neo*) into the 3′UTR that was flanked by two lox*P* sites to enable later excision. The targeting vector was linearized with *Sal*I and electroporated into 129 Ola-derived embryonic stem (ES) cells followed by selection with G418 (0.3 mg/ml). Correctly targeted ES clones were selected by Southern blotting via restriction enzyme sites (*Bgl*II and *BamH*I) that had been engineered into the construct used for homologous recombination and were injected into C57BL/6 N blastocysts. Chimeras were generated and mated to C57Bl/6N mice. Germline transmission of the targeted *Dmd* allele was confirmed in the F1 generation of heterozygous females via PCR screening for the *neo* cassette as well as by Southern blot analysis. The *neo* cassette was then removed by crossing F1 mice with mice that ubiquitously expressed *Cre* recombinase [61]. The genotype of the transgenic *Dmd*^*EGFP*^ mice was verified via a three-primer PCR (Fig. 1b) that allows the distinction between heterozygous and homozygous animals: FW#1: 5′-TGA CTC CCA ATA GTG GCA ACC-3′, FW#2 5′-GAG CAA AGA CCC CAA CGA GA-3′, REV: 5′-CCA TGC GGG AAT CAG GAG TT-3′ (wild-type = 202 bp, *Dmd*^*EGFP*^ = 304 bp). All animal experiments were conducted in accordance with institutional guidelines for the care and use of laboratory animals as approved by the local authorities (LaGeSo Berlin, T 0222/13).

### Isolation of *EDL* myofibers, culture, and differentiation of single fiber-derived satellite cells

*Extensor digitorum longus* (EDL) muscles were dissected from the hind limbs of 8-month-old *Dmd*^*EGFP*^ mice and their wild-type littermates and digested in 0.2 % collagenase type I (Sigma-Aldrich, Germany) in DMEM [62]. Muscles were then transferred to DMEM-filled horse serum (HS)-rinsed dishes using a heat-polished Pasteur glass pipette. Muscles were triturated until the fibers had separated, transferred to a fresh dish, and incubated at 37 °C, 5 % CO_2_. Intact fibers were transferred into fresh DMEM-filled and HS-rinsed dishes using a thinly bored Pasteur pipette and under a stereomicroscope to exclude any cell debris. The isolated myofibers with attached satellite cells (SC) were either fixed for immunofluorescence (IF) staining or cultured in DMEM supplemented with 20 % FBS (Sigma-Aldrich), 10 % HS (Sigma-Aldrich), and 1 % chicken embryo extract (CEE Ultrafiltrate, VWR International, Germany) in dishes that had been coated with 10 % Matrigel® (BDBiosciences, Germany) [63]. After 2 days, SC-derived myogenic progenitors started to migrate off the myofibers and formed myogenic colonies composed of proliferating myoblasts that differentiated into myotubes by days 6–8. This process was enhanced by changing the growth medium to low serum medium (4 % HS) after 5 days. Differentiating myoblasts were monitored daily under the microscope and fixed after 8 days for subsequent IF staining.

### Histological analysis

*Tibialis anterior* (TA), EDL, *quadriceps* (QUAD), *gastrocnemius* (GAS), and *soleus* (SOL) muscles, as well as the diaphragm (DIA), heart, stomach, *colon*, *ileum*, *duodenum*, brain, and eyes were dissected from 8–12-week-old (adult) or 10-month-old (aged) male *Dmd*^*EGFP*^ mice and from their age- and sex-matched wild-type littermates. The tissue was mounted on Tissue-Tek® O.C.T. (Hartenstein, Germany), frozen in fluid nitrogen cooled isopentane and processed for cryostat sectioning; 8-μm cross sections were collected from the mid-belly of the muscles and either stored at −80 °C or directly fixed and stained. Hematoxylin/eosin staining was done according to standard procedures.

Immunohistochemistry was performed on 8-μm cryosections using the *i*VIEW-Ventana ABC Kit (Ventana, AZ, USA) with primary antibodies and corresponding biotinylated secondary antibodies using the diaminobenzidine (DAB) visualization method. In order to suppress unspecific background staining, we used the MOM kit (Vector Laboratories, Burlingame, CA, USA) for all primary mouse antibodies. All primary and secondary antibodies used in this study are described in Additional file 2: Tables S1 and S2.

Immunofluorescent staining was performed on single fibers, differentiated myoblasts, and on 8-μm cryosections from different tissues. Fibers and myoblasts were fixed in 4 % PFA, washed, incubated 1 h at room temperature in blocking solution containing 5 % normal goat serum (NGS), 0.5 % BSA, and 0.2 % Triton X-100, followed by overnight incubation with primary antibodies. The cryosections were fixed in ice-cold acetone or 4 % PFA, washed in PBS, blocked, and incubated with primary antibodies for 1 h at room temperature or at 4 °C overnight. After primary antibody incubation, the samples were washed and incubated for 1 h with fluorescently labeled secondary antibodies (AlexaFluor® 488 and 568, Life Technologies, CA, USA, dilution 1:400), washed in PBS, and mounted in DAPI-containing mounting medium (Vectashield, Vector Laboratories, USA).

Primary antibodies in this study were directed against the following proteins or protein subdomains: laminin, β-spectrin, dystrophin, Dys2, MANDYS19, H4, GFP, FLAG, GFAP, CD31, α-, β-, and γ-sarcoglycan, α-dystroglycan, nNOS, and vinculin. Acetylcholine receptors were stained using AlexaFluor568 coupled α-bungarotoxin. Images were recorded with an inverted fluorescent microscope (Leica DMI4000).

### Serum CPK analysis

Blood was collected from the facial vein of 8-week-old wild-type, *Dmd*^*EGFP*^ and *mdx*-mice (*n* = 3 of each). Serum creatine phosphokinase (CPK) activity was measured with the Creatine Kinase Activity Assay Kit (Sigma-Aldrich, Munich, Germany) according to the manufacturer’s instructions.

### Morphometric analysis

8 μm cryosections of the EDL and *soleus* muscles from 8-week-old wild-type and *Dmd*^*EGFP*^ mice were stained with an anti-laminin antibody using the DAB visualization method to delineate the muscle fibers. Bright field color images were taken at ×200 magnification and saved as TIF files. The minimal Feret diameter was calculated for 500–820 fibers per muscle using the Image-Pro Plus v.7.0 software (Media Cybernetics, Rockville, MD, USA). Fibers at the border of the image were excluded from analysis. Three animals per group were analyzed, and the fiber diameters for EDL and *soleus* were plotted as a histogram (Fig. 3D).

### Western blot analysis

Protein was extracted from the *tibialis anterior* muscle or from the brain of 8-week-old male *Dmd*^*EGFP*^ mice and wild-type littermates using a combination of following two buffers (1:1) containing proteinase inhibitor cocktail: (i) 250 mM sucrose, 10 mM Tris-HCl, pH 8.2 and (ii) 20 % SDS, 20 % glycerol, 10 % β-mercaptoethanol, 12.5 % Western blot running buffer in bidest water; 50, 5, 0.5, or 0.05 μg protein were loaded on 3–8 % Tris-acetate gradient gels (Novex, Life Technologies), electrophoresed, and wet-blotted onto a nitrocellulose membrane. NuPage® LDS sample buffer (Invitrogen) was used for gel electrophoresis. The blots were probed using the iBind Flex® Western System (Invitrogen, LifeTechnologies) with mouse antibodies directed against the dystrophin rod domain (Dys1 or MANDYS19) and a rabbit polyclonal antibody against the dystrophin C-terminus (clone H4, self-made, gift from Cyrille Vaillend) together with secondary fluorescently labeled antibodies (AlexaFluor® 700 and 800). A second blot was probed with a monoclonal anti-GFP antibody together with a secondary fluorescent antibody (AlexaFluor® 700). As a loading control, a mouse monoclonal anti-vinculin antibody was used. Bands were visualized using the Odyssey CLx system (Li-Cor) (Fig. 1c). All used antibodies are listed in Additional file 2: Tables S1 and S2.

## Results and discussion

### Generation and validation of the *Dmd*^*EGFP*^ reporter mice

For generation of the *Dmd*^*EGFP*^ mice, the murine dystrophin locus on the X-chromosome was modified using a targeting vector (Fig. 1a). This resulted in a modification of dystrophin exon 79 whose natural termination codon was removed and a C-terminal fusion protein generated with a FLAG- and an EGFP-L221K coding sequence. The 3′UTR further contained a lox*P* flanked neomycin (*neo*) cassette for ES-cell selection (Fig. 1a). After germline transmission, the *neo* cassette was successfully removed by transient crossbreeding with ubiquitous Cre-deleter mice. *Dmd*^*EGFP*^ transgenic animals could be identified by allele-specific PCR (Fig. 1b). Hemizygous males, as well as hetero- and homozygous females were viable and fertile and were analyzed in comparison with their wild-type littermates.

An important consideration for our targeting strategy was the site for EGFP insertion into the endogenous dystrophin locus. The aim was (i) to target the major dystrophin isoforms that are expressed in most of the relevant tissues and (ii) to find out whether the natural endogenous dystrophin-EGFP expression would be strong enough for detection without further amplification by immunofluorescence. Since the FLAG-EGFP tag was appended to the C-terminus of dystrophin, to a protein domain where several interacting proteins bind, we had to investigate (iii) whether the genetic manipulation might cause a dystrophic phenotype per se and whether (iv) dystrophin and its binding partners from the DAPC would have the correct subcellular localization. (v) Finally, we wanted to investigate whether the published data in the literature on tissue-specific dystrophin expression patterns would correspond to those seen in our *Dmd*^*EGFP*^ mice.

The aim of our study was to generate a reporter mouse, in which dystrophin expression can be visualized and tracked in different tissues by means of EGFP fluorescence. Dystrophin and its numerous isoforms and splice variants share the C-terminal domain, which is very important for the interaction of the protein with its binding partners and might thus interfere with proper function; however, previous studies had already shown that C-terminally EGFP-tagged mini- or micro-dystrophins in transgenic mice or cells would be functional [64–66]. Therefore, we inserted the FLAG-EGFP sequence downstream of exon 79, the last exon present in the major dystrophin isoforms (Additional file 1: Figure S1). Insertion of the FLAG-EGFP downstream of exon 79, would theoretically tag the C-terminus and express the following proteins as fusions: Dp427 (B, M, P), Dp260, Dp140, Dp116, Dp71, Dp71d, and Dp71c. The alternative hydrophobic C-terminus of Dp71f and Dp71_Δ110_ would not be targeted in our model since skipping of exon 78 shifts the reading frame of the last exon 79 due to alternative splicing (Additional file 1: Figure S1) [31, 67]. In addition, Dp40 expressing another alternative C-terminus would not be tagged as well.

**Fig. 2:**
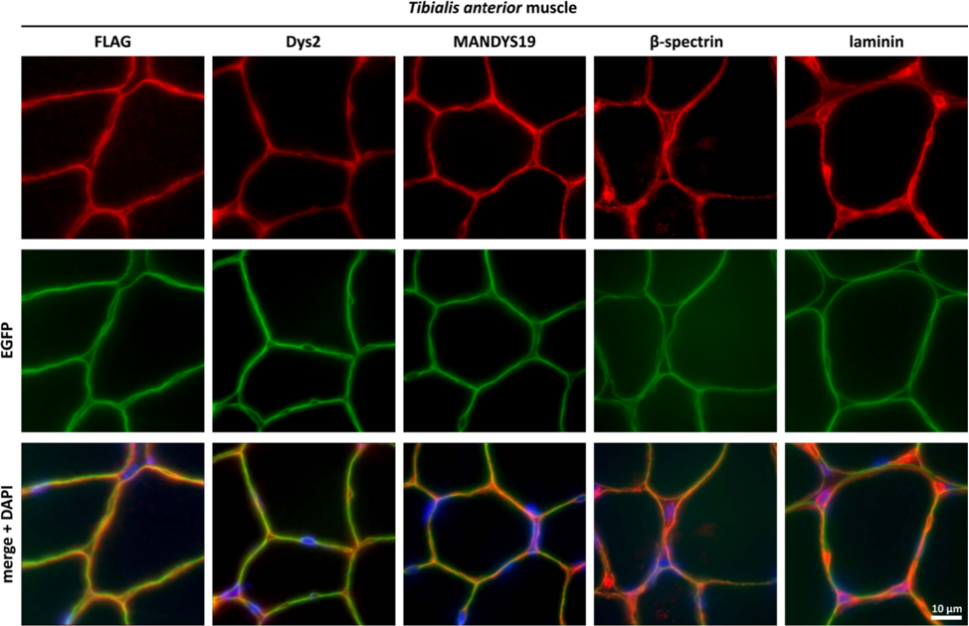
Correct localization of the EGFP-tagged dystrophin at the sarcolemma. Immunofluorescent staining of cryosections from the *tibialis anterior* of *Dmd* ^*EGFP*^ mice with antibodies against the FLAG-tag, C-terminal dystrophin (Dys2), rod-domain dystrophin (MANDYS19), cytoskeletal β-spectrin, and the basement membrane protein laminin (all colored in *red*). Exact colocalization was observed between the natural EGFP fluorescence (*green*) and the signals deriving from the two anti-dystrophin antibodies. Merged images were counterstained with DAPI (*blue*)

In order to investigate the expression pattern of the fusion protein, we first analyzed the skeletal muscle of *Dmd*^*EGFP*^ mice. The presence of the dystrophin-EGFP fusion protein was confirmed by Western blot analysis (Fig. 1c) using dystrophin antibodies against the rod and C-terminal domains in TA protein lysates. The bands from wild-type dystrophin and from dystrophin-EGFP had similar expression intensities as detected by the rod-domain-specific Dys1 antibody (Fig. 1c). Moreover, using MANDYS19 as another rod-domain-specific antibody, we compared dystrophin expression in protein extracts from the TA muscle of normal controls and of *Dmd*^*EGFP*^ mice over 3 orders of magnitude and found comparable protein concentrations of the wildtype Dp427 and the Dp427-EGFP band (Additional file 1: Figure S12). However, the band intensity of the *Dmd*^*EGFP*^ samples was lower if the C-terminal-specific (H4, Fig. 1c) antibody was used. A possible explanation could be a reduction of binding affinity of the H4 antibody through steric hindrance by the FLAG-EGFP tag. A similar difference was found in the Western blot of brain samples (Additional file 1: Figure S13). The molecular sizes of the proteins detected in both wild-type and *Dmd*^*EGFP*^ samples were comparable since differences in molecular weight of 27.9 kDa between full-length dystrophin and dystrophin-EGFP would not be easily detectable on Western blot at molecular weights beyond 400 kDa. We detected an EGFP band in the molecular size range of dystrophin only in the muscle and brain of *Dmd*^*EGFP*^ mice (Fig. 1c, Additional file 1: Figure S13B). Furthermore, the strong expression of dystrophin-EGFP could be directly observed under the microscope via the natural green fluorescence localized at the sarcolemma (Fig. 1d), while the EGFP signal showed exact superposition with the immune signal from the anti-FLAG antibody on TA sections (Fig. 2, first column).

### Dystrophin-EGFP expression in skeletal muscle and normal muscle morphology in *Dmd*^*EGFP*^ reporter mice

The correct localization of native EGFP fluorescence was further confirmed in TA, *soleus*, and *gastrocnemius *muscles by co-immunostaining with anti-dystrophin antibodies against the rod (MANDYS19) and C-terminal (Dys2) domains (Fig. 2, Additional file 1: Figure S2), which showed an exact superposition of the signals. MANDYS19 binds to the amino acid sequence encoded by dystrophin exons 21-22 and thus recognizes only the full-length protein [68]. All the alternative dystrophin promoters that control the expression of shorter dystrophin isoforms are located downstream of intron 29, and dystrophin isoforms generated from these promoters would not be picked up by the MANDYS19 antibody. Hence, the signal from the MANDYS19 antibody, which was similar to the signal of the Dys2 C-terminal antibody, confirmed the correct tagging and subcellular localization of the skeletal muscle Dp427 isoform. Correct expression of the ECM component laminin as well as of the subsarcolemmal β-spectrin was also confirmed on skeletal muscle sections (Additional file 1: Figure S3), where β-spectrin colocalized with dystrophin-EGFP in the myofibers.

Importantly, a comparison between the EGFP and laminin signals exhibited, as expected, distinct differences, with EGFP being present at the cytoplasmatic side of the sarcolemma and excluded from the capillaries. Notably, the endothelial cells of the endomysial capillaries were also stained by the anti-β-spectrin antibody where dystrophin-EGFP expression was absent. This suggests that dystrophin expression in the vascular system is confined to larger vessels that possess a *tunica media* (e.g., arteries and larger veins) [69].

**Fig. 3:**
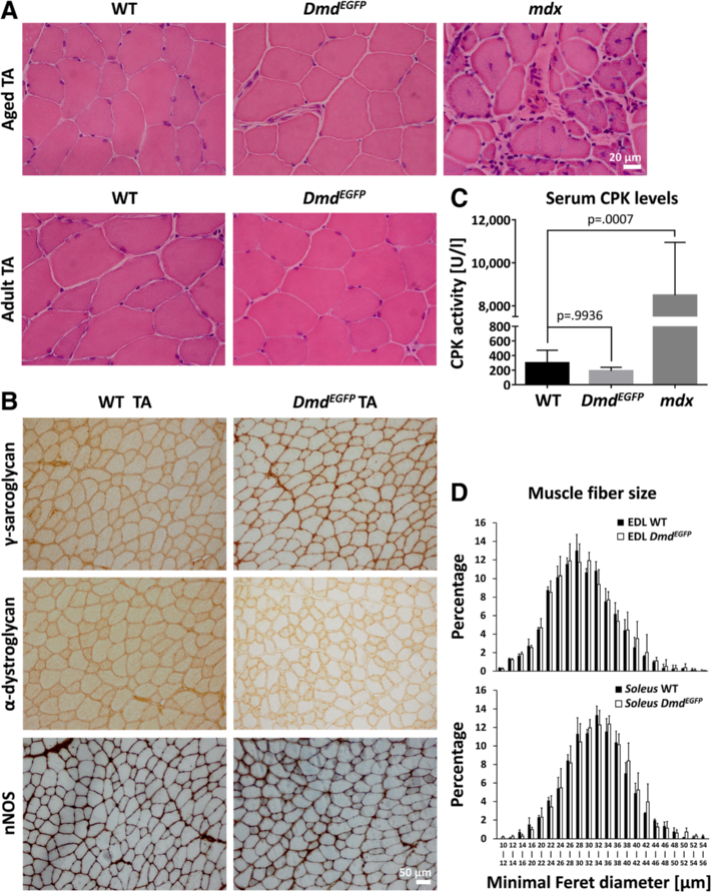
*Dmd* ^*EGFP*^ mice do not show signs of muscle dystrophy. **a** Hematoxylin and eosin (H&E) staining of *tibialis anterior* (TA) muscle sections from adult (8- to 12-week-old) and aged (10-month-old) wild-type (WT) and *Dmd* ^*EGFP*^ mice show normal morphology. For comparison, H&E staining of an aged-matched *mdx* TA muscle shows the morphological characteristics of muscle dystrophy comprising a large variation of fiber size, mononuclear cell infiltrates, fibrosis, and abundant centrally located myonuclei. **b** Wild-type and *Dmd* ^*EGFP*^ mice show normal localization and expression of the DAPC components α-dystroglycan, γ-sarcoglycan, and nNOS in TA muscle cross sections. **c** Serum creatine phosphokinase (CPK) activities in the serum of wild-type, *Dmd* ^*EGFP*^, and *mdx* mice (*n* = 3 of each genotype). Values are shown as means ± S.E.M., *p* values were calculated using one-way ANOVA. **d** The histograms depict the minimal Feret diameter of muscle fibers from EDL muscles (*upper panel*) and *soleus* muscles (*lower panel*) from adult wild-type and *Dmd* ^*EGFP*^ mice. Values are shown as means ± S.E.M. (*n* = 3 mice from each genotype were analyzed)

The C-terminal domain of dystrophin plays an important role in the binding and assembly of the DAPC, which has a crucial role for muscle function and sarcolemmal stability [70]. Adding a 27.9-kDa EGFP tag to the C-terminus of dystrophin might hence interfere with proper protein folding, its subcellular localization, and protein function, thereby provoking a muscular dystrophy such as seen in the *mdx* mouse model for Duchenne muscular dystrophy (DMD). We thus investigated the skeletal muscle phenotype. H&E staining of cross sections from different skeletal as well as from heart muscle did not show any differences between the adult reporter mice and their wild-type littermates (Fig. 3a, Additional file 1: Figure S4). Moreover, no dystrophic changes could be detected in 10-month-old *Dmd*^*EGFP*^ mice, which were well beyond the age when dystrophic changes are commonly encountered in *mdx* mice. For comparison, we analyzed aged-matched *mdx* mice, which exhibited strong signs of fibrosis, immune cell infiltration, and central nucleation—all hallmarks of an advanced dystrophic process (Fig. 3a). Thus, histological analysis confirmed that EGFP tagging of the dystrophin C-terminus did not cause overt muscle pathology. This notion was further confirmed by measurement of normal CPK activities in the serum of *Dmd*^*EGFP*^ mice, which were largely elevated only in the *mdx* mice (Fig. 3c). Moreover, analysis of fiber size (minimal Feret diameters) of EDL and soleus muscles from *Dmd*^*EGFP*^ in comparison to wild-type mice did not reveal any significant differences (Fig. 3d).

Finally, we screened different skeletal muscles for the expression of representative members of the various DAPC sub-complexes. We confirmed normal sarcolemmal/subsarcolemmal expression patterns for α-, β-, γ-sarcoglycan, α-dystroglycan, as well as for nNOS suggesting proper function of the fusion protein (Fig. 3b, Additional file 1: Figures S5-S9). We conclude that no deleterious changes of the dystrophin protein structure might have occurred that would disturb those interactions. However, for some components, it appeared as if the expression levels would differ between *Dmd*^*EGFP*^ and wild-type mice. However, the DAB-mediated visualization of immune signals is not really quantitative and subject to too many confounding factors in order to pick up subtle differences. Here, it would be preferable in the future to employ mass spectrometric techniques to identify and quantify proteins that bind to the dystrophin-EGFP, which could be easily immunoprecipitated with an anti-FLAG or anti-GFP antibody [71].

### Dystrophin-EGFP expression in cardiac and smooth muscle

**Fig. 4:**
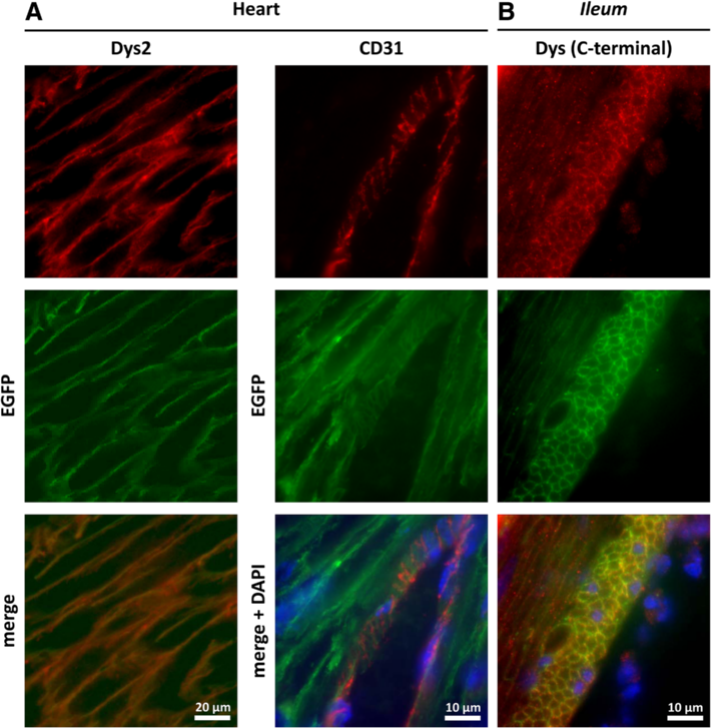
Dystrophin-EGFP expression in heart and smooth muscle. **a** Colocalization at the sarcolemma of the immunofluorescent signal from dystrophin (Dys2) and natural EGFP fluorescence (*green*) in heart muscle. Blood vessel endothelium is stained with anti-CD31 (*red*) and also exhibits, at least, partial colocalization with the EGFP signal. **b** Immunofluorescent staining with a C-terminal rabbit anti-dystrophin antibody (*red*) of *ileum* sections verifies colocalization with natural EGFP fluorescence (*green*). Sections were counterstained with DAPI (*blue*)

Dystrophin expression in the heart is controlled by the muscle- and brain-specific promoters, while expression in the smooth muscles of the gastrointestinal (GI) tract is controlled by the muscle-specific promoter. We confirmed correct dystrophin-EGFP expression in the heart (Fig. 4a) as well as in the *ileum *(Fig. 4b) as a representative part of the GI tract through verification of colocalization between the natural EGFP signal and signals generated by anti-dystrophin antibodies. Moreover, intramuscular cardiac blood vessels showed an EGFP signal as well, which partially colocalized with the anti-CD31 signal serving as a marker for endothelial cells (Fig. 4a, right column). Dystrophin is known to be expressed in the walls of large arteries and veins as well as in small arteries [69], in all those vessels that are able to regulate their vascular tone through contraction of the smooth muscle cells in their *tunica media*. In contrast, dystrophin was absent from small veins (not shown), whose diameter is only passively regulated by the blood volume and elastic fibers.

The localization of dystrophin near the plasma membrane of smooth muscle cells via its natural EGFP fluorescence could be readily observed in cryosections of other intestinal organs as well. In cross sections of stomach, *ileum*, and *duodenum *of *Dmd*^*EGFP*^ mice, we observed bundles of longitudinal and circular layers of smooth muscle cells with dystrophin located at their periphery (Additional file 1: Figure S10A). The distribution of the EGFP signal at the membrane of smooth muscle cells was discontinuous, which is in line with previous studies using dystrophin antibodies [24]. This phenomenon is due to the exclusion of dystrophin from the *adherens* junctions between smooth muscles [72].

### Dystrophin-EGFP expression in non-muscle tissue

**Fig. 5:**
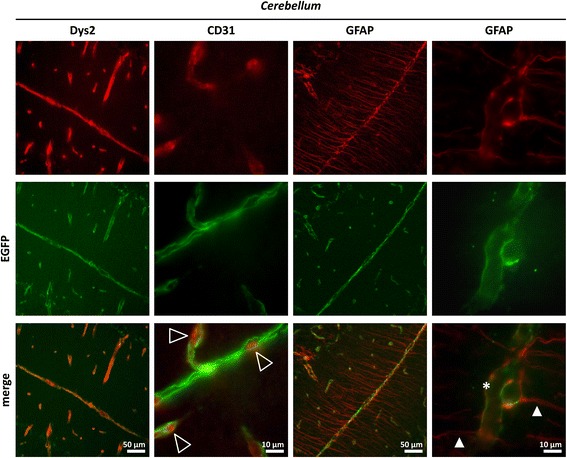
Dystrophin-EGFP expression in the brain. Immunofluorescent staining of brain sections with the Dys2 antibody (*red*) shows colocalization of the natural EGFP signal (*green*) with the dystrophin signal in the *cerebellum* of *Dmd* ^*EGFP*^ mice. Partial colocalization of the EGFP signal with the anti-CD31 (*red*) signal confirmed the presence of dystrophin expression also in the blood vessels of the brain (*second column*). Open arrowheads show the EGFP signal surrounding the signal from the CD31 marker. Immunofluorescent staining with anti-glial fibrillary acidic protein (GFAP, *red*) shows colocalization with the EGFP signal at the glial endfeet in the *cerebellum *(*third column*). A higher magnification of the anti-GFAP signal is shown in the *fourth column*. EGFP expression is confined to the glial endfeet (*asterisk*), whereas the green fluorescent tag is not expressed in the glial cell body (*closed arrowheads*)

In order to investigate whether the different dystrophin isoforms were tagged in non-muscle tissues as well, we analyzed the EGFP expression in the brain and retina of *Dmd*^*EGFP*^ mice. Western blot analysis of whole brain lysates from *Dmd*^*EGFP*^ mice and wild-type littermates (Additional file 1: Figure S13) revealed only very low levels of the full-length dystrophin Dp427 isoform using the rod domain-specific Dys1 antibody. Similar low levels of Dp427 could be detected with an anti-GFP antibody in *Dmd*^*EGFP*^ samples. The Dp140 and Dp71 isoforms could be detected with the C-terminal specific H4 antibody only in wild-type but not in *Dmd*^*EGFP*^ samples, which further confirms our observation from Western blot analysis on TA lysates that the FLAG-EGFP-tag causes steric hindrance against binding of the H4 antibody. In the *Dmd*^*EGFP*^ samples, the corresponding bands, which run at a higher molecular weight (of 170 and 100 kDa, respectively), were detected using the anti-GFP antibody, confirming the correct targeting of the Dp427, Dp140, and Dp71 isoforms in the brain of the *Dmd*^*EGFP*^ mice. Furthermore, we confirmed the natural EGFP expression in cryosections of the brain where the EGFP signal showed exact colocalization with the immune signal from staining with an anti-dystrophin antibody (Fig. 5, left column).

**Fig. 6:**
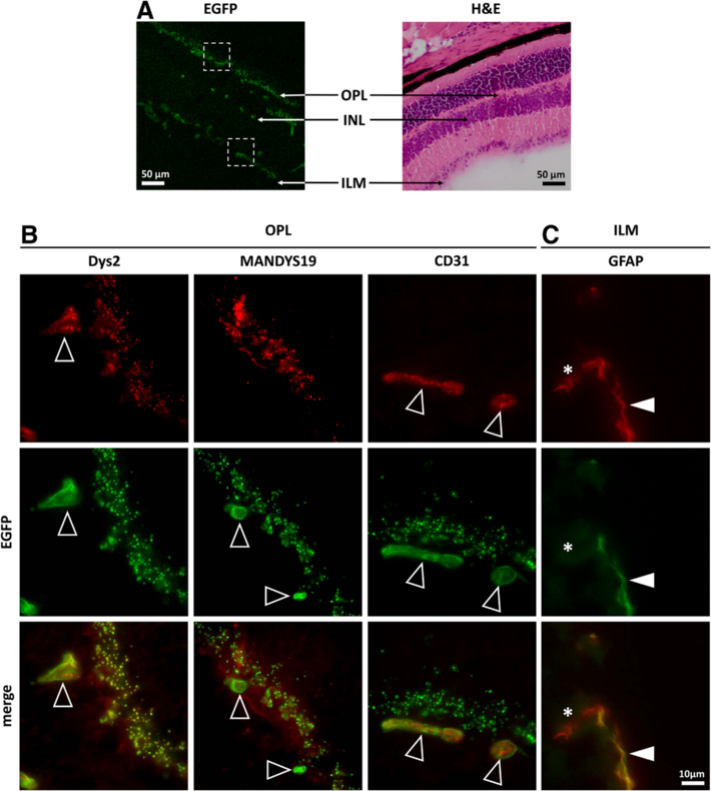
Dystrophin-EGFP expression in the retina. **a** Presence of the natural EGFP signal in the different layers of the retina: outer plexiform layer (OPL), inner nuclear layer (INL), and inner limiting membrane (ILM) (*left image*). These regions are depicted on a corresponding H&E stained cross section from the retina of a wild-type mouse (*right image*). A higher magnification of the EGFP-positive regions highlighted by the squares in **a** is presented in **b** and **c**. **b** Immunofluorescent staining with antibodies against dystrophin (Dys2, MANDYS19, *red*) and CD31 (*red*) showed differential colocalization of natural EGFP signal (*green*) in the retinal OPL of *Dmd* ^*EGFP*^ mice. *Arrows* indicate EGFP-positive retinal blood vessels that were detected with Dys2 and anti-CD31 antibody, but not with the rod-specific antibody. Strong punctuated expression of the natural EGFP was observed in the OPL region corresponding to the photoreceptor terminals. In this region, an exact superposition with the Dys2 signal and some overlay with the MANDYS19 signal were observed, whereas no anti-CD31 signal was detected. **c** Immunofluorescent staining with antibodies against the glial fibrillary protein (GFAP, *red*) showed partial colocalization with the natural EGFP signal at the ILM. *Arrowheads* indicate colocalization between GFAP and EGFP at the glial endfeet. The *asterisk* marks the GFAP positive region, which did not express EGFP, corresponding to the glial cell body

In the retina of *Dmd*^*EGFP*^ reporter mice, EGFP expression was observed at the outer plexiform layer (OPL), the inner limiting membrane (ILM), and sporadically at the inner nuclear layer (INL) (Fig. 6a). A strong punctate EGFP signal colocalized with dystrophin immunofluorescence and was detectable in the OPL of the retina (Fig. 6b). Exact colocalization of the natural EGFP signal was detected when using a C-terminal anti-dystrophin antibody (Dys2). However, the rod domain-specific MANDYS19 antibody did not recognize all EGFP-positive structures confirming the targeting of different isoforms in the retina. Although it is widely accepted that dystrophin is expressed at the photoreceptor terminals [25], a recent in-depth study of dystrophin expression in the retina suggested the presence of dystrophin also in the OPL layer, where rod and cone bipolar cells of the ON-type are located. Of note, the authors could not exclude artifacts by fluorescent signals from neighboring regions [53]. Hence, using the *Dmd*^*EGFP*^ mice, a better characterization of this region and the distribution of dystrophin in the retina could be achieved without the use of antibodies and potentially associated staining artifacts.

In the brain and retina of *Dmd*^*EGFP*^ mice, we observed natural dystrophin-EGFP expression around blood vessels that were stained with the anti-CD31 endothelial cell marker (Figs. 5 and 6b). Moreover, staining with an antibody against the glial fibrillary acidic protein (GFAP) revealed partial colocalization of the dystrophin-EGFP with the Bergmann glia in the *cerebellum *(Fig. 5), the glial cells of the *hippocampus* (Additional file 1: Figure S10B), and the Müller cells at the inner limiting membrane of the retina (Fig. 6c). In these regions, the EGFP signal only colocalized with the glial endfeet, which confirms a characteristic expression pattern of the Dp71 isoform [73]. On the other hand, dystrophin is also expressed in the blood vessels, mainly in the wall surrounding the endothelial cells. The EGFP signal was at some locations exactly encircling the CD31 immunosignal (Fig. 5), suggesting expression of the dystrophin in the outer walls of the vessels, but not in the vascular endothelium. However, from our immunofluorescent images, it did not become entirely clear, whether the green fluorescence really derived from the vessel wall or from the glial endfeet. Here, high-resolution imaging techniques like STED microscopy could resolve the issue.

It was reported in the literature that the full-length dystrophin isoform in the brain was expressed in neurons such as in Purkinje cells of the *cerebellum* or in neurons of the *hippocampus* [21, 74, 75]. Unfortunately, we were unable to detect any natural EGFP fluorescence in the aforementioned regions using our standard fluorescence EGFP-imaging techniques. However, via immunostaining using an anti-GFP antibody, we detected neuronal dystrophin-EGFP expression in CA1/CA2 regions of the *hippocampus *and in Purkinje cells (Additional file 1: Figure S11). The apparent absence of natural EGFP fluorescence in the neurons of *Dmd*^*EGFP*^ mice could be explained by the much lower expression levels of the full-length Dp427 (B) dystrophin in the brain in contrast to the Dp71 isoform, which gave much stronger signals in the Western blot analysis (Additional file 1: Figure S13). Moreover, immunofluorescence staining with an anti-GFP antibody shows large differences in the GFP signal intensities between Dp71-expressing blood vessels and Dp427-expressing neurons. For the detection of dystrophin-EGFP in the neurons, we had to use high exposure times that led to massive overexposure of the EGFP-positive blood vessels (Additional file 1: Figure S11). Even after immunostaining with an anti-GFP antibody, using lower exposure times to image the blood vessels would fail to visualize the neurons.

### EGFP fluorescence is detectable on the level of single muscle fibers and in satellite cell-derived myotubes

**Fig. 7:**
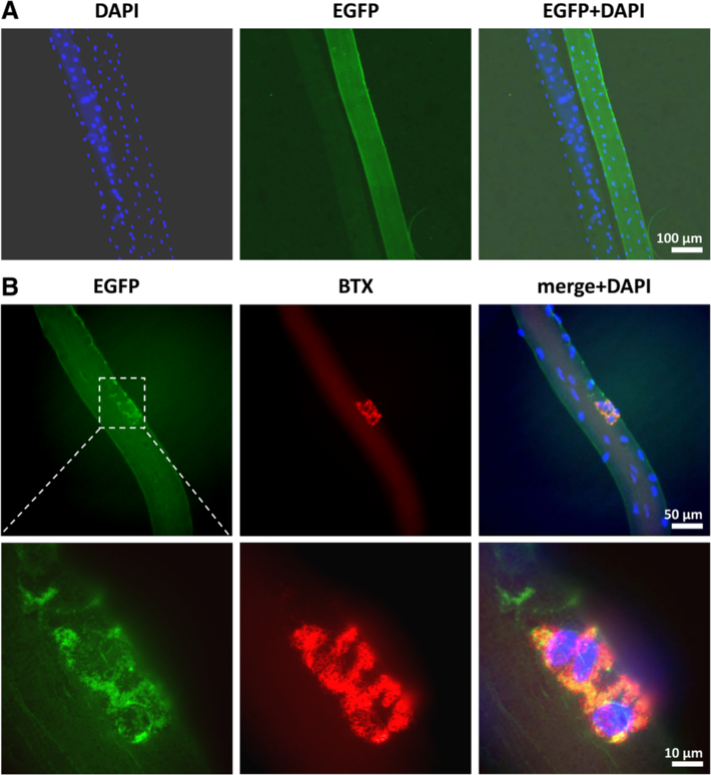
Dystrophin-EGFP expression of single fibers. **a** Isolated single EDL muscle fibers from *Dmd* ^*EGFP*^ mice and wild-type littermates. The natural EGFP fluorescence (*green*) permits easy distinction between *Dmd* ^*EGFP*^ and wild-type fibers without necessity for immunostaining. Fibers were fixed and stained with DAPI (*blue*). **b** Natural EGFP expression at the neuromuscular junction of a single fiber co-stained with AlexaFluor568 labeled α-bungarotoxin (BTX, *red*). The higher magnification shows colocalization of natural dystrophin EGFP with the acetylcholine receptor. Fibers were counterstained with DAPI

On the level of single myofibers, we wanted to explore whether the presence of dystrophin-EGFP would allow live-cell imaging of dystrophin expression in cultures of isolated myofibers. Indeed, freshly isolated myofibers from *Dmd*^*EGFP*^ mice were strongly EGFP-fluorescent and stood in stark contrast to the dark wild-type fibers (Fig. 7a). In addition, strong dystrophin-EGFP expression was detected at the neuromuscular junction (NMJ) of single fibers that had been co-stained with AlexaFluor568-labeled α-bungarotoxin, as a specific marker for acetylcholine receptors (Fig. 7b). Hence, the strong EGFP expression at the NMJ makes our model suitable for longitudinal in vivo studies on the dynamics of dystrophin at the NMJ.

**Fig. 8:**
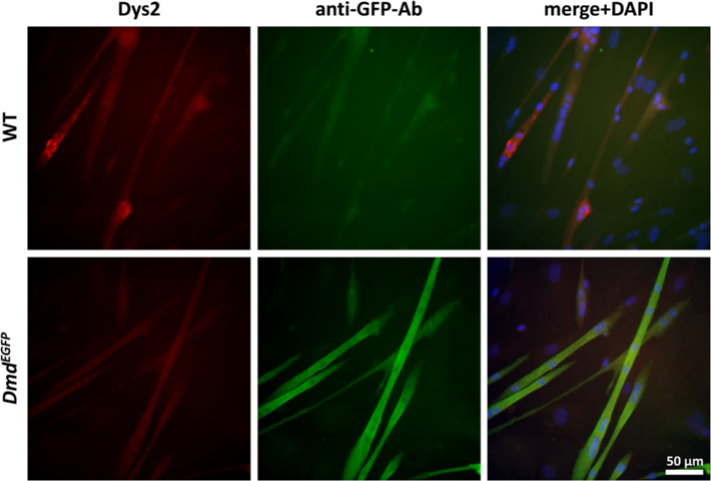
Dystrophin-EGFP expression of satellite cell-derived myotubes. Dystrophin-EGFP expression of in vitro differentiated satellite cells; isolated single fibers with their attached satellite cells from wild-type (WT) and *Dmd* ^*EGFP*^ mice were cultured for 4 days and differentiated for a further 8 days. Forming myotubes were fixed and stained with anti-GFP (*green*) and Dys2 (*red*) antibodies and counterstained with DAPI (*blue*)

Finally, we wanted to find out whether freshly isolated satellite cells cultured in vitro would express dystrophin once they formed myotubes. Unfortunately, after 8 days of culture, the natural EGFP signal in the myotubes was too weak to be picked up by epi-fluorescence microscopy, possibly due to the still too low numbers of available dystrophin molecules. Interestingly, using a well-established anti-GFP antibody (Additional file 2: Table S1), the dystrophin-EGFP fusion protein was detected with a much stronger signal then using any of the anti-dystrophin antibodies (Fig. 8). This further confirms the usefulness of our animal model to study dystrophin expression during myogenesis or muscle regeneration. The ability of ex vivo satellite cells from *Dmd*^*EGFP*^ reporter mice to produce dystrophin EGFP once differentiating could serve as a valuable tool to be exploited for transplantation and cell tracing experiments.

## Conclusion

This study describes and validates a *Dmd*^*EGFP*^ reporter mouse that was generated through a C-terminal dystrophin-EGFP fusion protein, which is expressed from its natural promoters. The addition of the tag did not have any influence on the structure and function of the protein and the assembly of the dystrophin-associated protein complex. The natural EGFP expression is strong enough in the skeletal, heart, and smooth muscles, brain, and retina allowing detection of dystrophin even on the single fiber level without the need for immunofluorescent staining. We confirmed the successful targeting of the major dystrophin isoforms that express the last exon 79. The mouse model will provide better insight into the normal dystrophin expression in the muscle as well as in the non-muscle tissue and will facilitate studies on the dynamics of dystrophin expression. Furthermore, the crossing of the *Dmd*^*EGFP*^ allele into the *mdx* background in *cis* (e.g., the *Dmd* exon 23 nonsense mutation lies on the same allele as the FLAG-EGFP tag) will provide an excellent model to investigate dystrophin re-expression in vivo or ex vivo after various gene therapy protocols that are aimed at the re-establishment of the dystrophin open reading frame or in naturally occurring revertant fibers.

## Abbreviations

bp, base pair; BTX, bungarotoxin; CNS, central nervous system; CPK, creatine phosphokinase; DAPC, dystrophin-associated protein complex; DAPI, 4′,6-diamidino-2-phenylindole dihydrochloride; DG, dentate gyrus; DIA, diaphragm; DMD, Duchenne muscular dystrophy; ECM, extracellular matrix; EDL, *extensor digitorum longus* muscle; EGFP, enhanced green fluorescent protein; ES cells, embryonic stem cells; Ex, exon; GAS, *gastrocnemius*; GFAP, glial fibrillary acidic protein; H&E, hematoxylin and eosin staining; HS, horse serum; IF, immunofluorescence; ILM, inner limiting membrane (of the retina); INL, inner nuclear layer (of the retina); kbp, kilobase pair, kDa, kilo-Dalton; *neo*, neomycin; NMJ, neuromuscular junction; nNOS, neuronal nitric oxide synthase; OPL, outer plexiform layer (of the retina); QUAD, *quadriceps muscle*; SOL, *soleus *muscle; TA,* tibialis anterior* muscle

## Additional files

Additional file 1: Figures S1-S13.
**Figure S1.** Targeting of various dystrophin isoforms in *Dmd* ^*EGFP*^ mice. A schematic representation of known dystrophin isoforms and splice variants and their alternative C-termini; in *Dmd* ^*EGFP*^ mice, the FLAG-EGFP sequence is fused to the exon 79 coding sequence of *Dmd*. We expected successful targeting of the isoforms Dp427 (M, B, P), Dp260, Dp140, Dp116, Dp71, Dp71d, and Dp71c. The alternatively spliced variants of Dp71, namely Dp71f, Dp71_Δ110_, and Dp40 contain an alternative C-terminus that does not contain the coding sequence of exon 79 and hence cannot be tagged with EGFP. Dark blue squares show the alternative C-terminal sequence generated by skipping of exon 78. Due to alternative splicing, the C-terminus of Dp40 is entirely different and fails to express EGFP as well. M, muscle-specific promoter; B, brain-specific promoter; P, Purkinje cell promoter; N, N-terminus; C, C-terminus of the polypeptide chain. **Figure S2.** Correct localization of the EGFP-tagged dystrophin at the sarcolemma. Immunofluorescent staining of cross sections from *soleus* (SOL), *gastrocnemius* (GAS), and *tibialis anterior* (TA) muscles of transgenic mice with anti-dystrophin antibodies specific to** (A)** the C-terminal domain (Dys2), **(B)** the rod domain (MANDYS19); all colored in *red*. Exact colocalization was observed between the natural EGFP fluorescence (*green*) and the signals deriving from the two anti-dystrophin antibodies. **Figure S3.** Correct localization of the EGFP-tagged dystrophin at the sarcolemma. Immunofluorescent staining of cross sections from *soleus *(SOL), *gastrocnemius *(GAS), and *tibialis anterior* (TA) muscles of transgenic mice with anti-dystrophin antibodies specific to **(A)** cytoskeletal β-spectrin and **(B)** the basement membrane protein laminin; all colored in red. **Figure S4.** Absence of a dystrophic phenotype in H&E stained skeletal and heart muscle of adult and aged wild-type (WT) and *Dmd* ^*EGFP*^ mice. Skeletal muscles *quadriceps* (QUAD), *gastrocnemius *(GAS), *extensor digitorum longus* (EDL), *soleus *(SOL), diaphragm (DIA), and heart muscle from adult wild-type and transgenic mice show normal morphology with consistency in fiber caliber and no evidence for the presence of central nuclei, fibrosis, or the presence of inflammatory cells. No further changes are observed in aged mice in the QUAD, GAS, EDL, and heart muscle. **Figure S5.** Immunohistochemistry of skeletal muscles of wild-type (WT) and *Dmd* ^*EGFP*^ mice with an antibody against α-sarcoglycan. Normal sarcolemmal expression of α-sarcoglycan can be observed in the following skeletal muscles: *gastrocnemius *(GAS), *soleus *(SOL), *tibialis anterior* (TA), *quadriceps *(QUAD), and *extensor digitorum longus* (EDL)*.* **Figure S6.** Immunohistochemistry of skeletal muscles of wild-type (WT) and *Dmd* ^*EGFP*^ mice with an antibody against β-sarcoglycan. Normal sarcolemmal expression of β-sarcoglycan can be observed in the following skeletal muscles: *gastrocnemius *(GAS), *soleus *(SOL), *tibialis anterior* (TA), *quadriceps *(QUAD), and *extensor digitorum longus *(EDL). **Figure S7.** Immunohistochemistry of skeletal muscles of wild-type (WT) and *Dmd* ^*EGFP*^ mice with an antibody against γ-sarcoglycan. Normal sarcolemmal expression of γ-sarcoglycan can be observed in the following skeletal muscles: *gastrocnemius *(GAS), *soleus *(SOL), * tibialis anterior *(TA), *quadriceps *(QUAD), and *extensor digitorum longus* (EDL)*.* **Figure S8.** Immunohistochemistry of skeletal muscles of wild-type (WT) and *Dmd* ^*EGFP*^ mice with an antibody against α-dystroglycan. Normal sarcolemmal expression of α-dystroglycan can be observed in the following skeletal muscles: *gastrocnemius *(GAS), *soleus *(SOL), *tibialis anterior *(TA), *quadriceps *(QUAD), and *extensor digitorum longus* (EDL). **Figure S9.** Immunohistochemistry of skeletal muscles of wild-type (WT) and *Dmd* ^*EGFP*^ mice with an antibody against nNOS. Normal sarcolemmal expression of nNOS can be observed in the following skeletal muscles: *gastrocnemius *(GAS), *soleus *(SOL), *tibialis anterior* (TA), *quadriceps *(QUAD), and *extensor digitorum longus *(EDL)*.* **Figure S10.** EGFP expression in the smooth muscle and in the *hippocampus*. **(A) **Sections from the stomach, *duodenum*, and *colon* of *Dmd* ^*EGFP*^ mice were stained with DAPI (*blue*). Strong natural EGFP expression (*green*) was detectable in the circular and longitudinal muscular layers. **(B)** Immunofluorescent staining of brain sections with an anti-glial fibrillary acidic protein antibody (GFAP, *red*) shows colocalization with the EGFP signal (*green*) at the glial endfeet in the *hippocampus*; counterstaining with DAPI (*blue*). A higher magnification is shown in the *right column*. **Figure S11.** EGFP expression in the *hippocampus *and in the *cerebellum*.** (A)** *Left*: Overview with DAPI staining of the mouse *hippocampus *with the CA1-3 regions and the dentate gyrus (DG). The dashed square is zoomed in on the *right side*. Neuronal cells (*asterisk*) of the *Dmd* ^*EGFP*^ mouse show much lower full-length dystrophin-EGFP expression than the blood vessels (*arrowhead*) as visualized by immunostaining with an anti-GFP antibody (*green*). Due to the high expression differences, the images are depicted with two different exposure times. **(B) **The same pattern can be observed in the *cerebellum *of *Dmd* ^*EGFP*^ mice stained with anti-GFP antibody (*green*). Dystrophin-EGFP expression in the Purkinje cells (*asterisks*) can be detected only with higher exposure times. All sections are counterstained with DAPI (*blue*). **Figure S12.** Western blot analysis of full-length dystrophin expression. A western blot of different amounts (50, 5, 0.5, 0.05 μg) of whole protein extracts from the TA muscle of wild-type and *Dmd* ^*EGFP*^-mice was probed with a dystrophin antibody against the rod domain (MANDYS19) and against vinculin as loading control. Both wild-type and transgenic mice show comparable dystrophin protein expression levels. **Figure S13.** Western blot analysis of dystrophin-EGFP expression in the brain. Western blot analysis of whole brain lysates using antibodies specific against the rod domain (Dys1) and the C-terminal domain (H4) of dystrophin, as well as an anti-GFP antibody. Two different contrast settings were used **(A, B)** in order to identify different dystrophin isoforms in the brain, which are expressed at different levels. **(A)** Dp71 is detected using the H4 antibody in the wild-type samples. The targeted isoform Dp71-EGFP shows a band running at 100 kDa detected by the anti-GFP antibody. **(B)** Higher contrast enhancement of the blot show Dp427 full-length dystrophin in wild-type and transgenic samples using the Dys1 antibody. The anti-GFP antibody also detected the targeted full-length form. Dp140 is detected using the H4 antibody in the wild-type samples. The targeted isoform Dp140-EGFP shows a band running at 170 kDa which is detected by the anti-GFP antibody. Vinculin served as loading control. WT, wild-type. (PDF 1438 kb) Additional file 2: Tables S1-S2.
**Table S1:** Primary antibodies. **Table S2:** Secondary antibodies. (DOC 70 kb)
