# Supplementary material for: Characterization of a DmdEGFP reporter mouse as a tool to investigate dystrophin expression
Source: Skelet Muscle. 2016 Jul 5;6:25. doi: 10.1186/s13395-016-0095-5 (PMC4932663; doi:10.1186/s13395-016-0095-5)
Supplement: Additional file 1: Figures S1-S13. — Figure S1. Targeting of various dystrophin isoforms in Dmd EGFP mice. A schematic representation of known dystrophin isoforms and splice variants and their alternative C-termini; in Dmd EGFP mice, the FLAG-EGFP sequence is fused to the exon 79 coding sequence of Dmd. We expected successful targeting of the isoforms Dp427 (M, B, P), Dp260, Dp140, Dp116, Dp71, Dp71d, and Dp71c. The alternatively spliced variants of Dp71, namely Dp71f, Dp71Δ110, and Dp40 contain an alternative C-terminus that does not contain the coding sequence of exon 79 and hence cannot be tagged with EGFP. Dark blue squares show the alternative C-terminal sequence generated by skipping of exon 78. Due to alternative splicing, the C-terminus of Dp40 is entirely different and fails to express EGFP as well. M, muscle-specific promoter; B, brain-specific promoter; P, Purkinje cell promoter; N, N-terminus; C, C-terminus of the polypeptide chain. Figure S2. Correct localization of the EGFP-tagged dystrophin at the sarcolemma. Immunofluorescent staining of cross sections from soleus (SOL), gastrocnemius (GAS), and tibialis anterior (TA) muscles of transgenic mice with anti-dystrophin antibodies specific to (A) the C-terminal domain (Dys2), (B) the rod domain (MANDYS19); all colored in red. Exact colocalization was observed between the natural EGFP fluorescence (green) and the signals deriving from the two anti-dystrophin antibodies. Figure S3. Correct localization of the EGFP-tagged dystrophin at the sarcolemma. Immunofluorescent staining of cross sections from soleus (SOL), gastrocnemius (GAS), and tibialis anterior (TA) muscles of transgenic mice with anti-dystrophin antibodies specific to (A) cytoskeletal β-spectrin and (B) the basement membrane protein laminin; all colored in red. Figure S4. Absence of a dystrophic phenotype in H&E stained skeletal and heart muscle of adult and aged wild-type (WT) and Dmd EGFP mice. Skeletal muscles quadriceps (QUAD), gastrocnemius (GAS), extensor digitorum longus (ED [file 13395_2016_95_MOESM1_ESM.pdf]

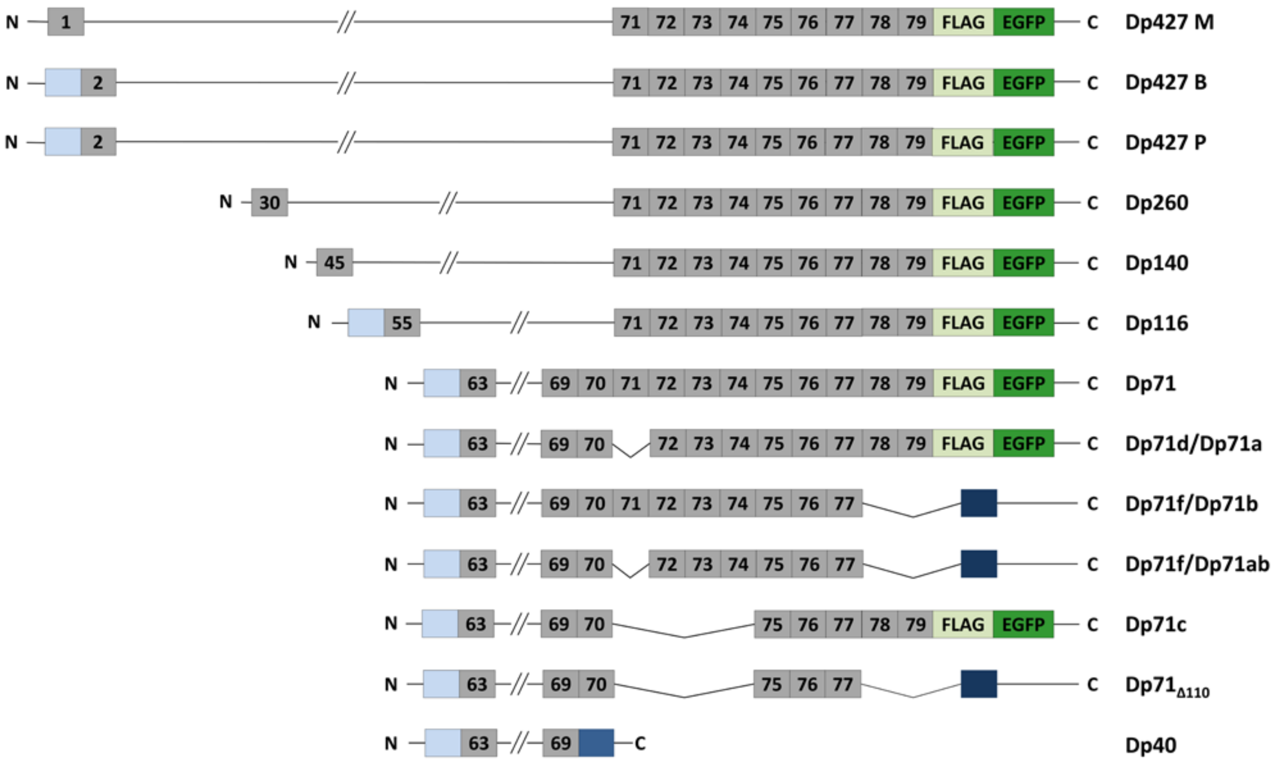
N 1 71 72 73 74 75 76 77 78 79 FLAG EGFP C Dp427 M
N 2 71 72 73 74 75 76 77 78 79 FLAG EGFP C Dp427 B
N 2 71 72 73 74 75 76 77 78 79 FLAG EGFP C Dp427 P
N 30 71 72 73 74 75 76 77 78 79 FLAG EGFP C Dp260
N 45 71 72 73 74 75 76 77 78 79 FLAG EGFP C Dp140
N 55 71 72 73 74 75 76 77 78 79 FLAG EGFP C Dp116
N 63 69 70 71 72 73 74 75 76 77 78 79 FLAG EGFP C Dp71
N 63 69 70 72 73 74 75 76 77 78 79 FLAG EGFP C Dp71d/Dp71a
N 63 69 70 71 72 73 74 75 76 77 C Dp71f/Dp71b
N 63 69 70 72 73 74 75 76 77 C Dp71f/Dp71ab
N 63 69 70 75 76 77 78 79 FLAG EGFP C Dp71c
N 63 69 70 75 76 77 C Dp71Δ110
N 63 69 C Dp40

Figure S1

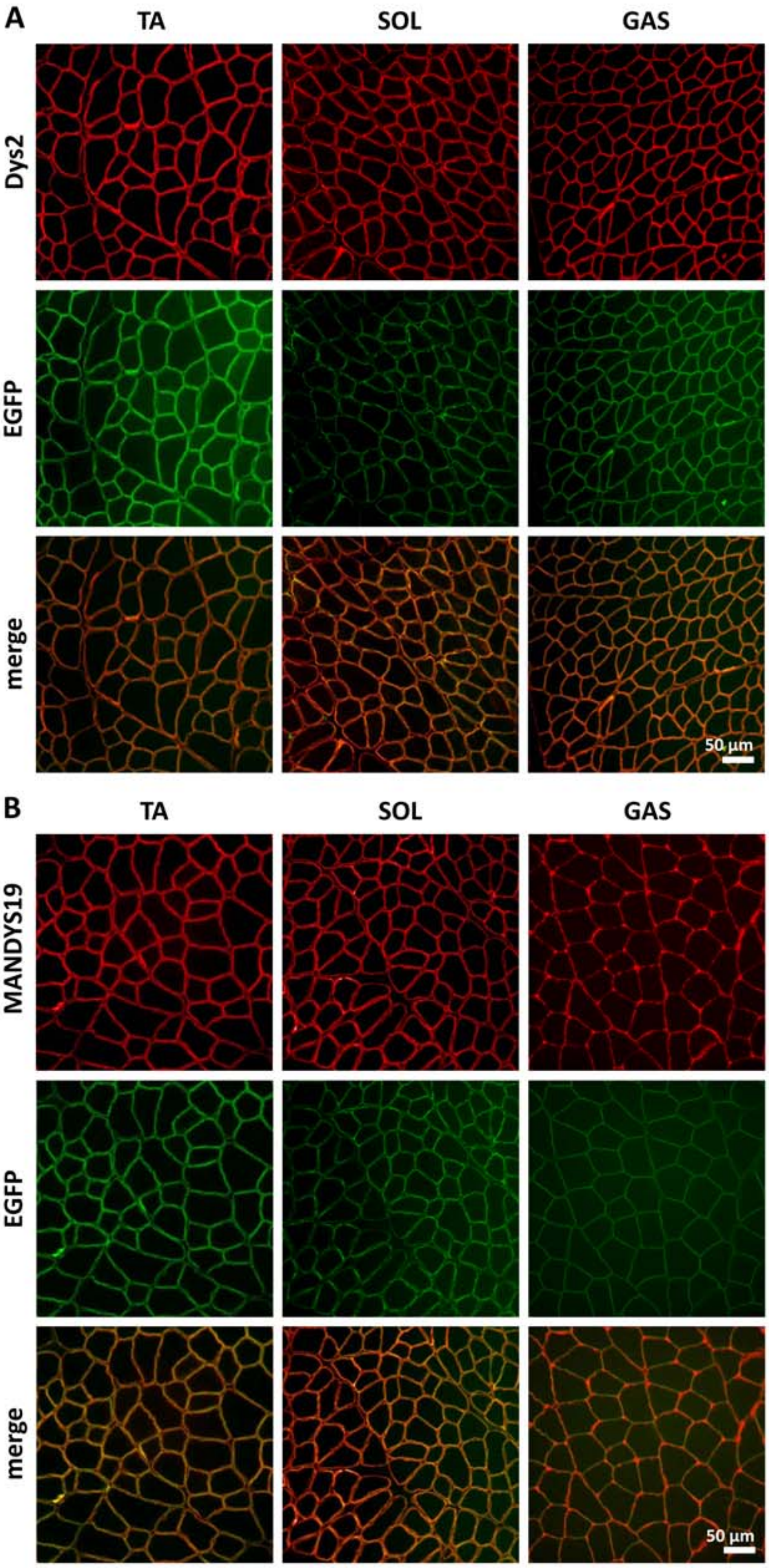
A
TA
SOL
GAS
Dys2
EGFP
merge
50 µm
B
TA
SOL
GAS
MANDYS19
EGFP
merge
50 µm

Figure S2

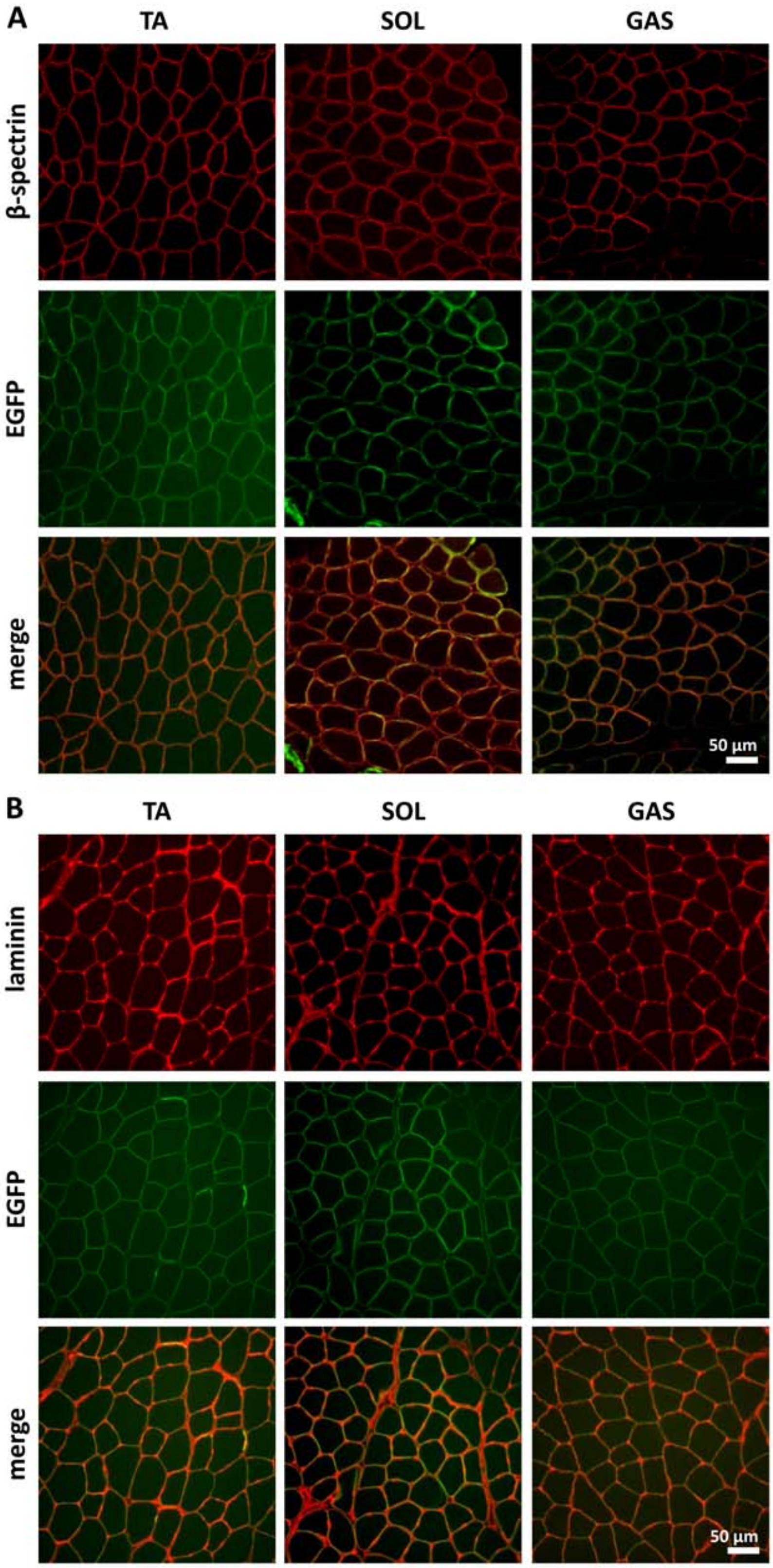
A
TA
SOL
GAS
β-spectrin
EGFP
merge
50 μm
B
TA
SOL
GAS
laminin
EGFP
merge
50 μm

Figure S3

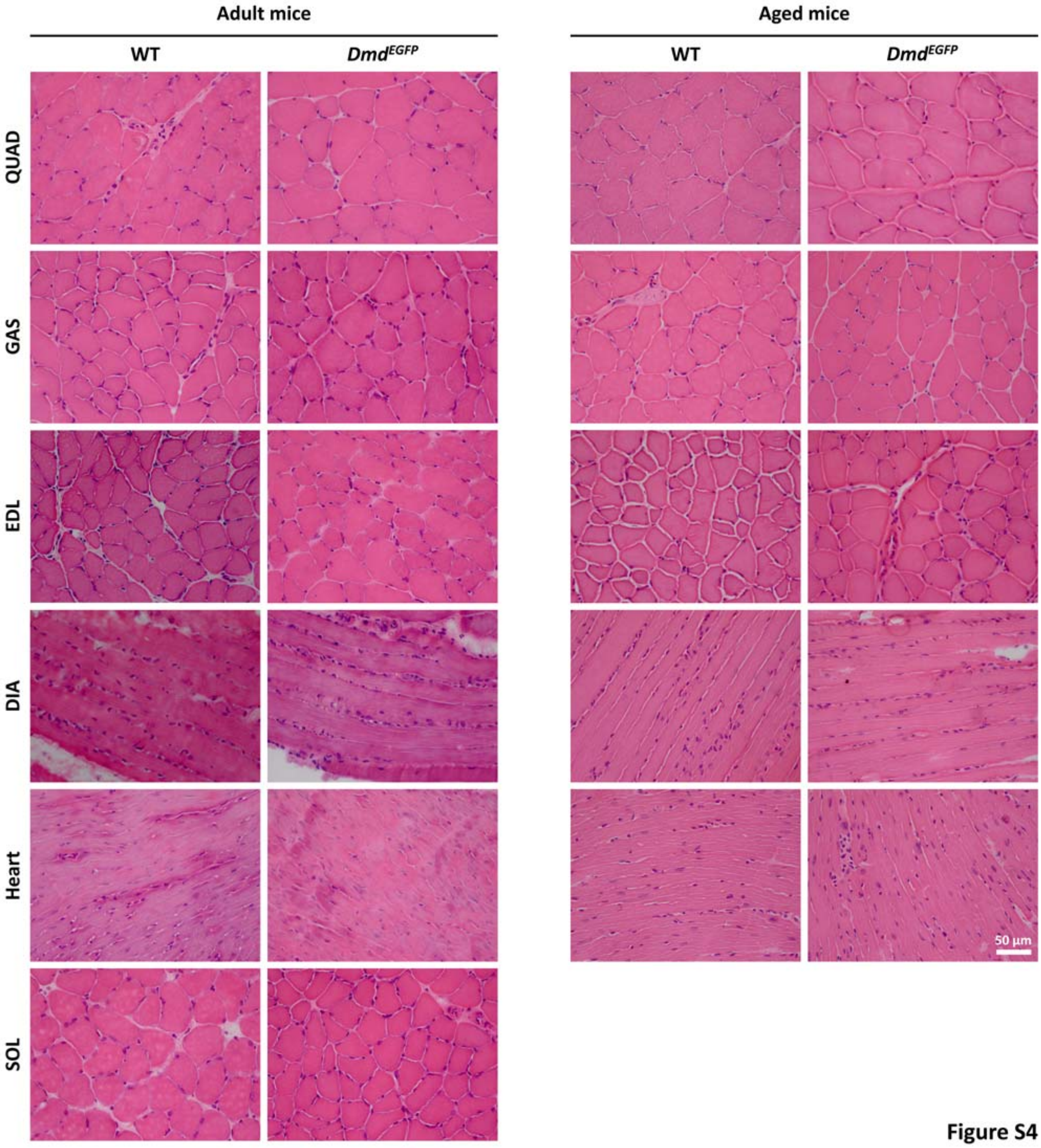

Adult mice
Aged mice
WT
DmdEGFP
WT
DmdEGFP
QUAD
GAS
EDL
DIA
Heart
SOL
50 µm

Figure S4

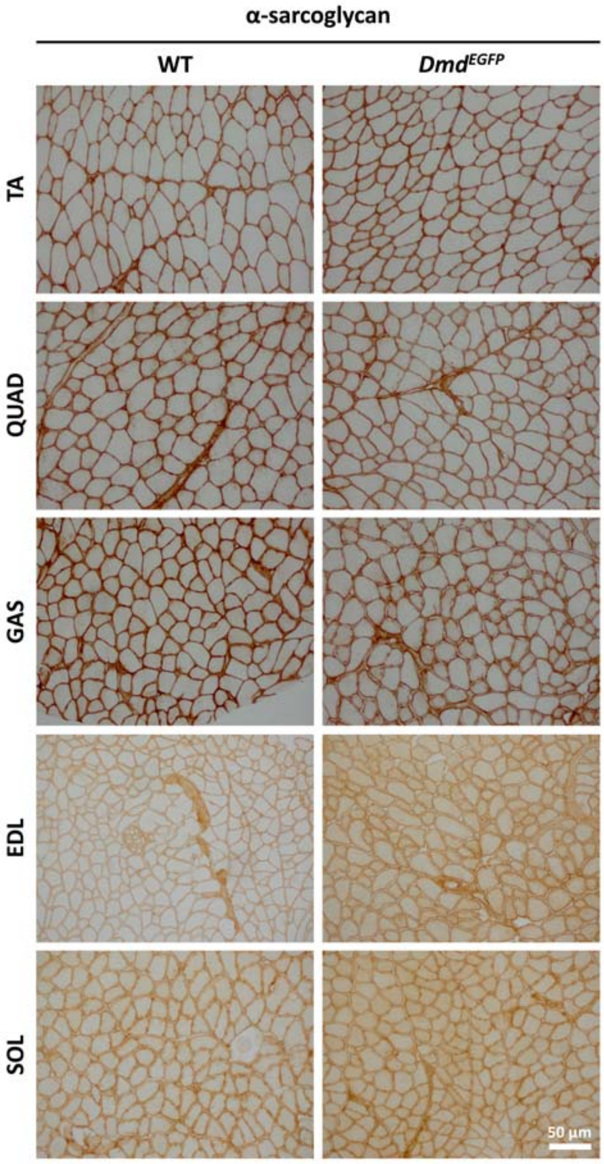

Figure S5

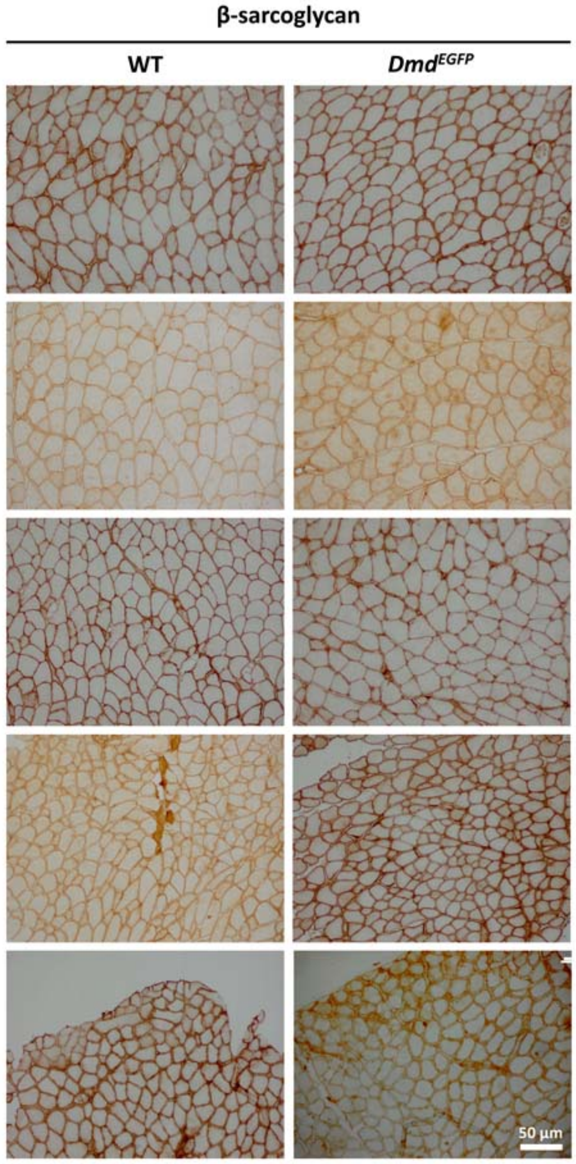

Figure S6

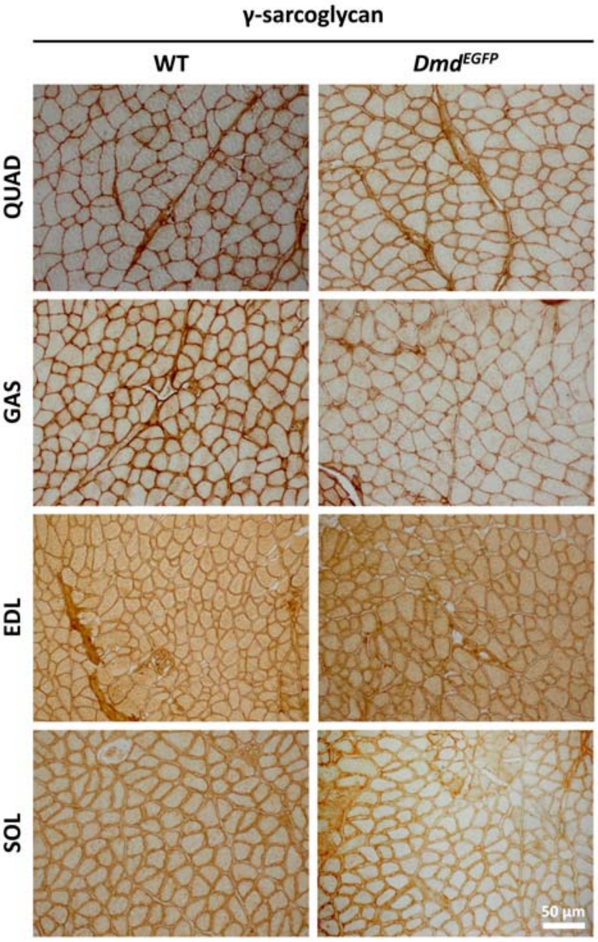

Figure S7

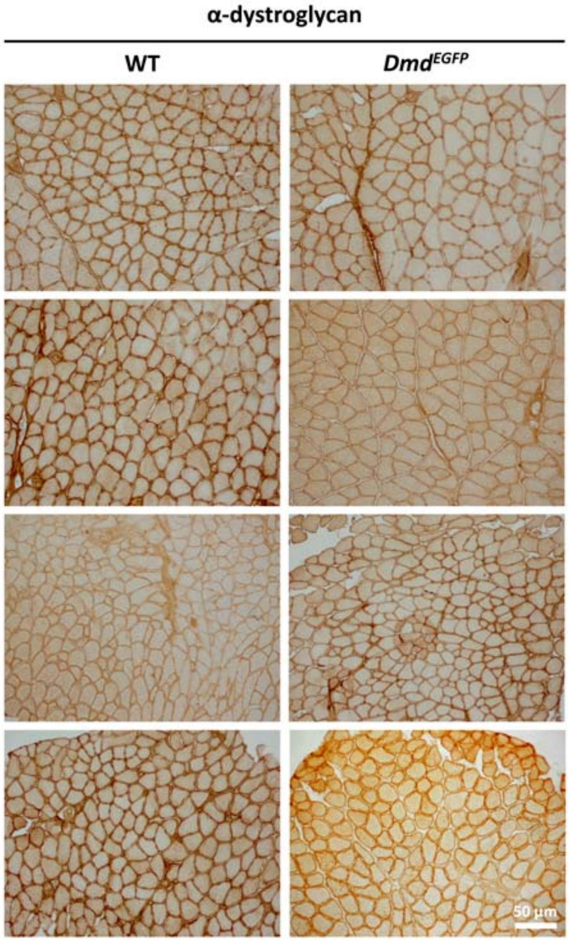

Figure S8

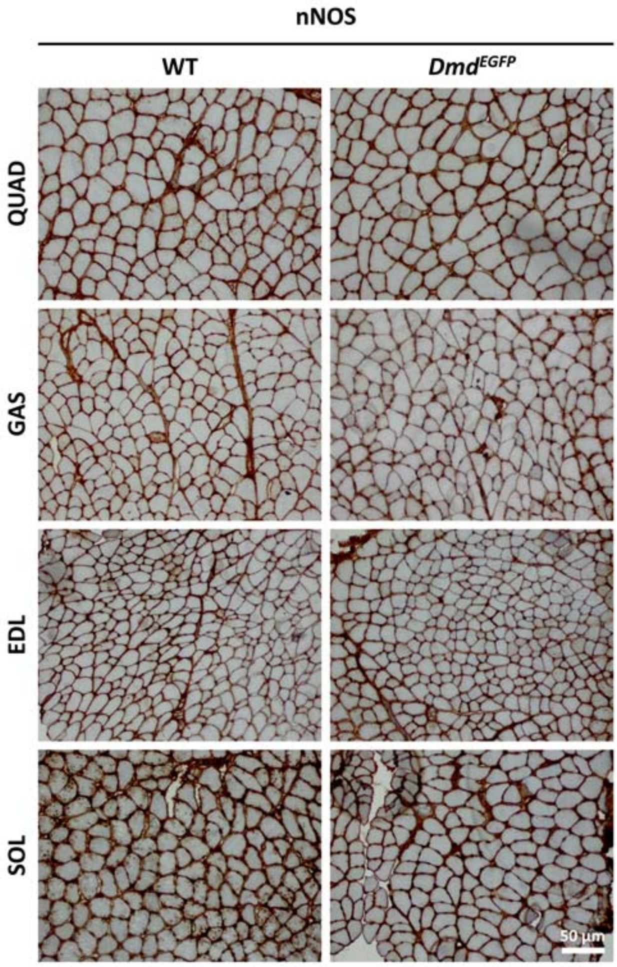

Figure S9

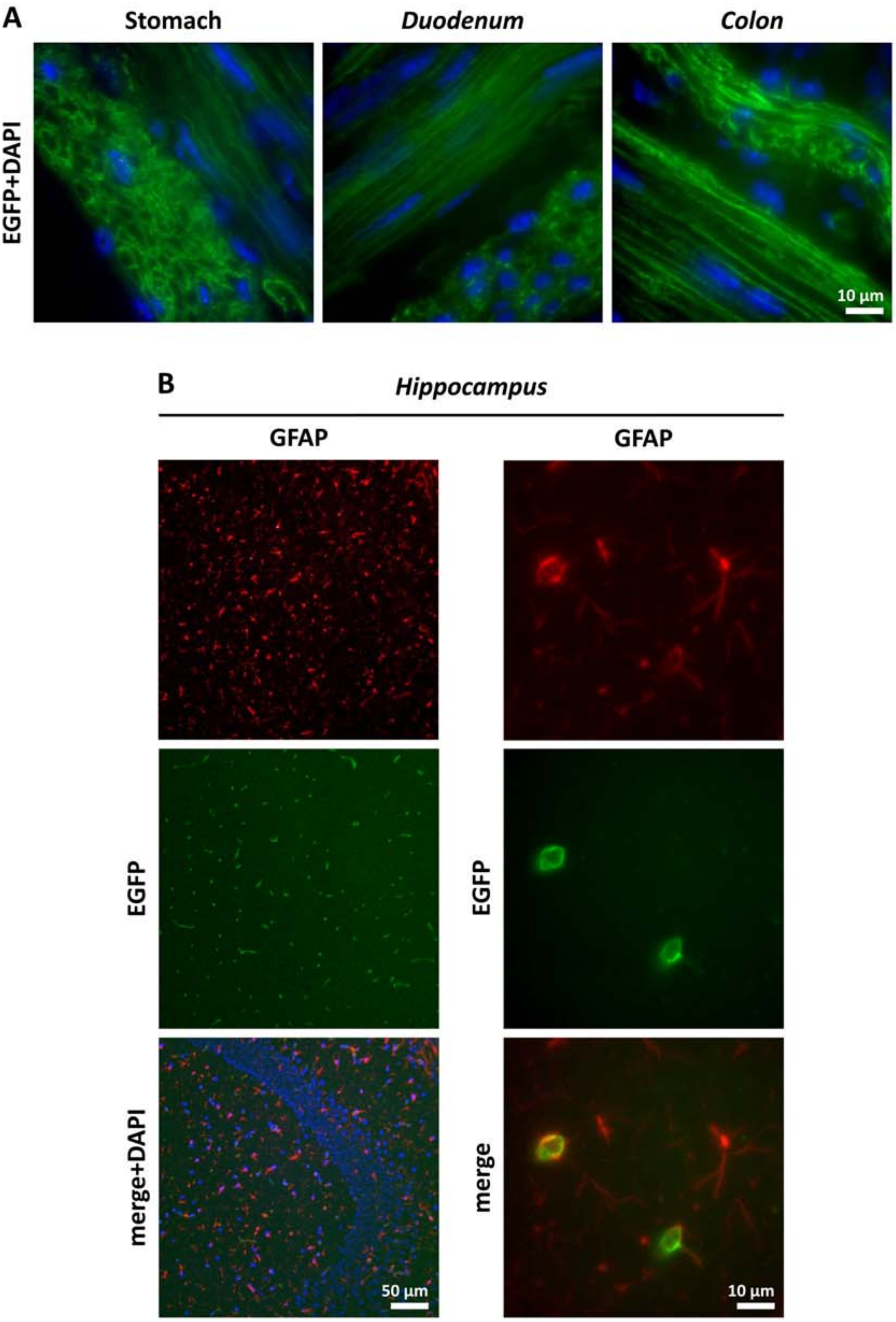
A
Stomach
Duodenum
Colon
EGFP+DAPI
10 µm
B
Hippocampus
GFAP
GFAP
EGFP
EGFP
merge+DAPI
merge
50 µm
10 µm

Figure S10

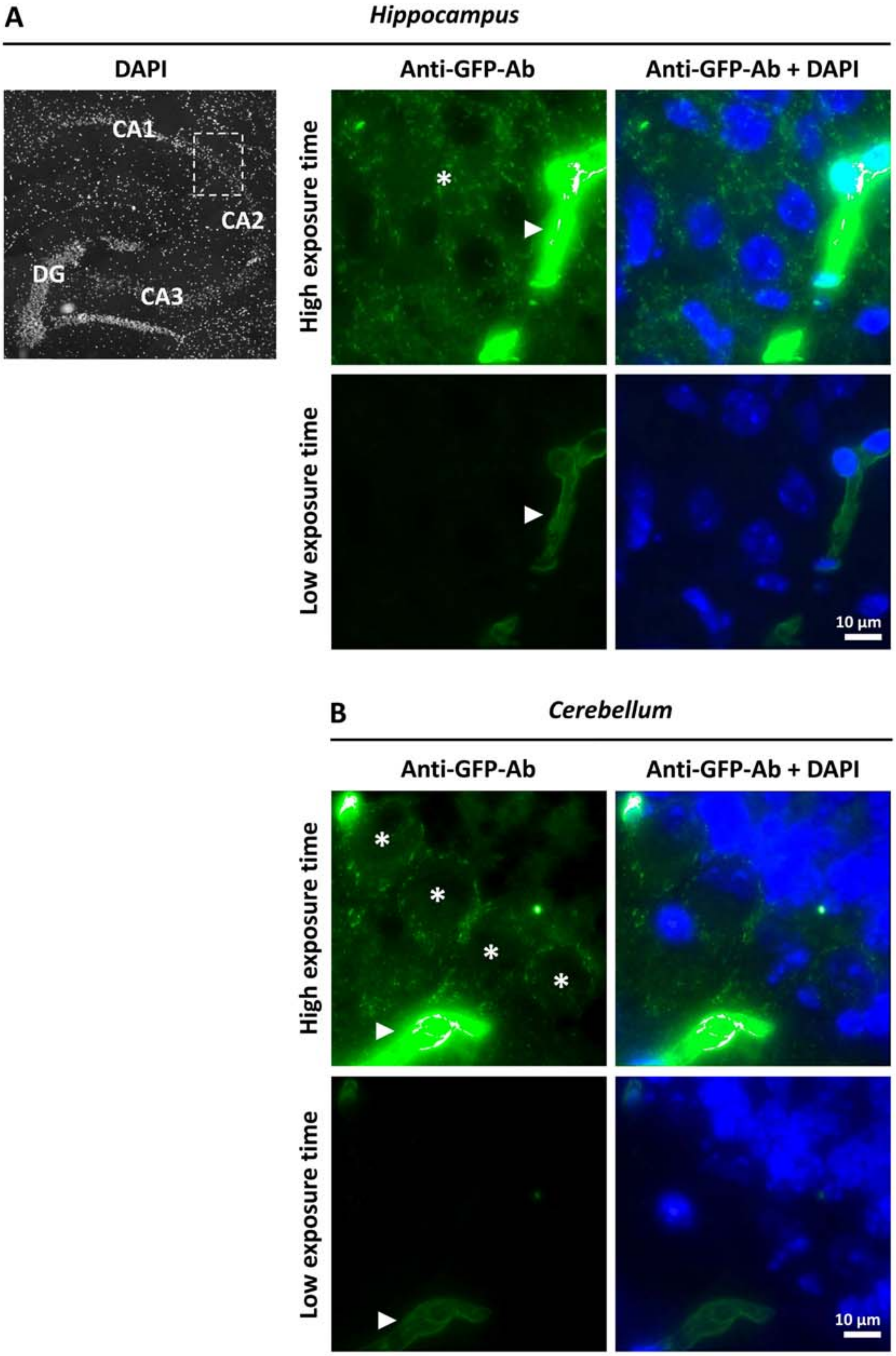
A
Hippocampus
DAPI
Anti-GFP-Ab
Anti-GFP-Ab + DAPI
CA1
CA2
DG
CA3
High exposure time
Low exposure time
10 µm
B
Cerebellum
Anti-GFP-Ab
Anti-GFP-Ab + DAPI
High exposure time
Low exposure time
10 µm

Figure S11

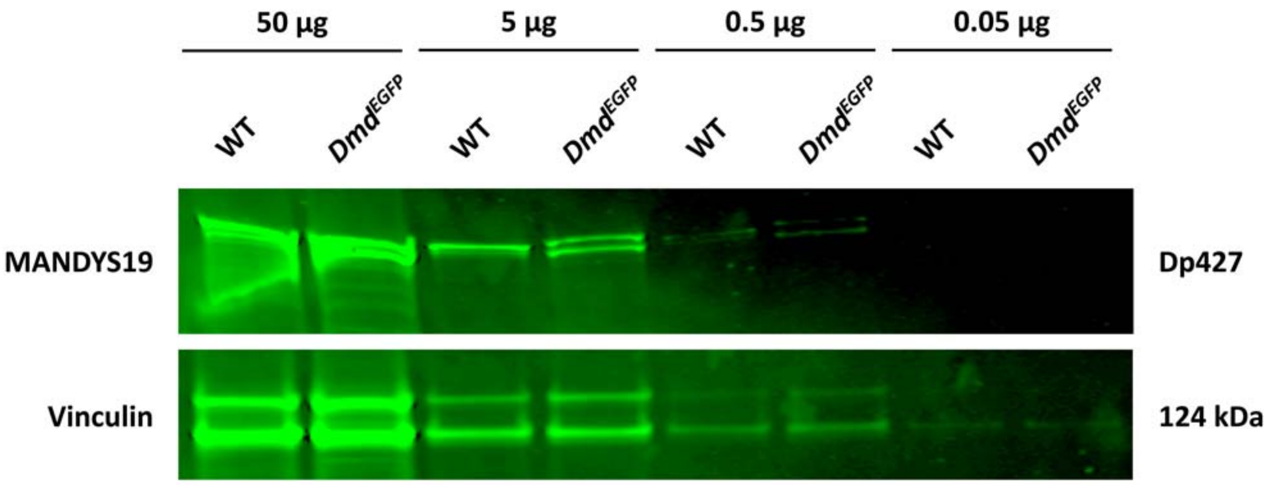
50 µg
5 µg
0.5 µg
0.05 µg
WT
Dmd^EGFP
WT
Dmd^EGFP
WT
Dmd^EGFP
WT
Dmd^EGFP
MANDYS19
Dp427
Vinculin
124 kDa

Figure S12

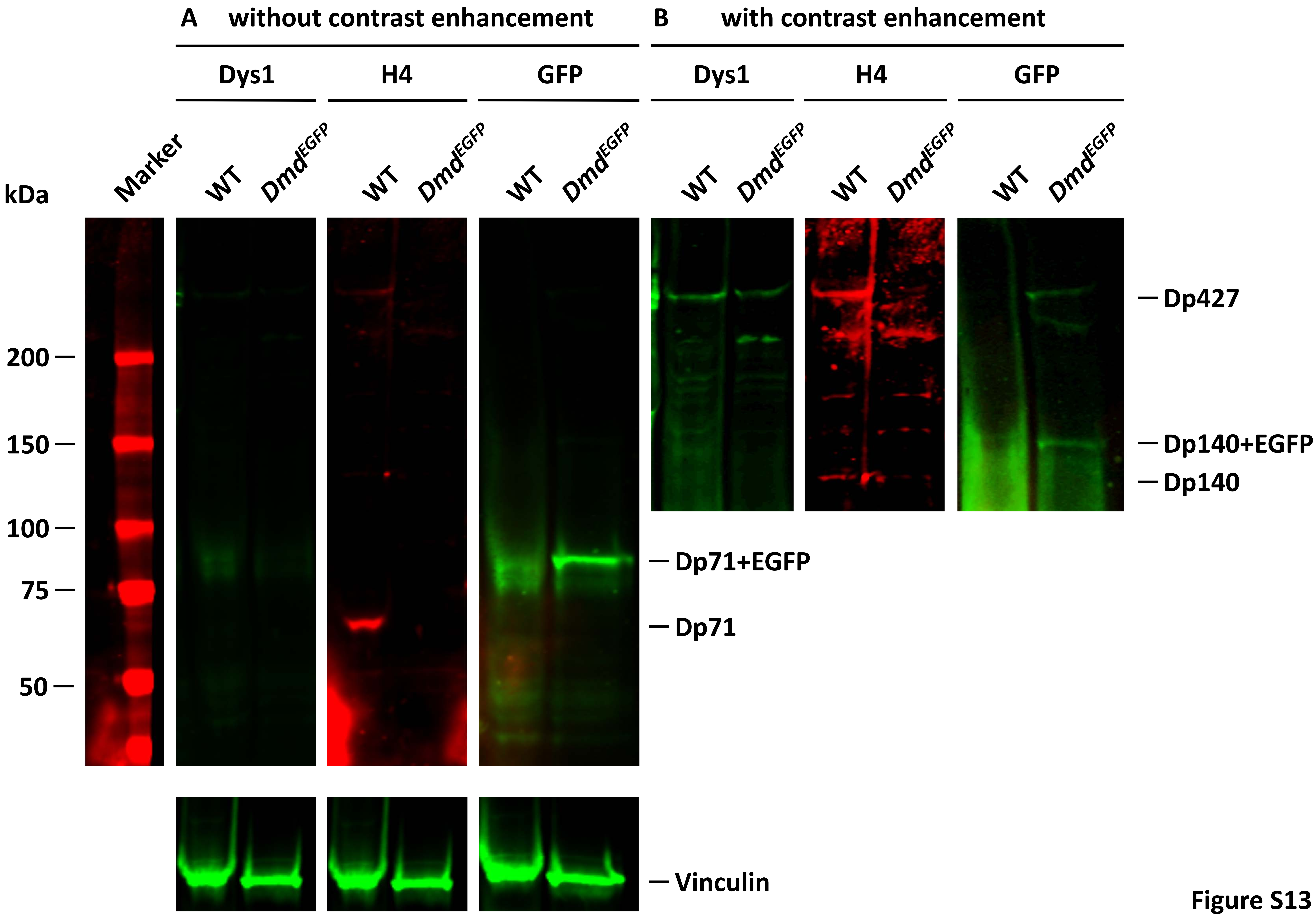
A without contrast enhancement
B with contrast enhancement
Dys1
H4
GFP
Dys1
H4
GFP
kDa
Marker
WT
DmdEGFP
200
150
100
75
50
Dp427
Dp140+EGFP
Dp140
Dp71+EGFP
Dp71
Vinculin

Figure S13
