# Supplementary material for: Characterization of a DmdEGFP reporter mouse as a tool to investigate dystrophin expression
Source: Skelet Muscle. 2016 Jul 5;6:25. doi: 10.1186/s13395-016-0095-5 (PMC4932663; doi:10.1186/s13395-016-0095-5)
Supplement: Additional file 2: Tables S1-S2. — Table S1: Primary antibodies. Table S2: Secondary antibodies. (DOC 70 kb) [file 13395_2016_95_MOESM2_ESM.doc]

**Additional file 2**

Table S1: Primary antibodies

| **Antigen** | **Isotype** | **Clonality** | **Species reactivity** | **Dilutions** | | | **Supplier** |
| --- | --- | --- | --- | --- | --- | --- | --- |
|  |  |  |  | **WB** | **IF** | **IHC** |  |
| α-actinin-2 | rabbit IgG | polyclonal | human, mouse, chicken, cow | 1:2,000 |  |  | Abcam, UK |
| α-bungarotoxin-CF^TM^ 568 |  |  |  |  | 1:500 |  | Biotium, Hayward, CA, USA |
| α-dystroglycan | mouse IgM | IIH6C4 | human, guinea pig, canine, rabbit, mouse, rat |  |  | 1:10 | Millipore, Darmstadt, Germany |
| CD31 | rabbit IgG | polyclonal | mouse, human, pig |  | 1:25 |  | Abcam, UK |
| dystrophin, C-terminal | rabbit IgG | polyclonal | mouse, rat, human |  | 1:100 |  | Thermoscientific, Wuppertal, Germany |
| dystrophin, rod domain (Dys1) | mouse IgG2a | monoclonal (Dy4/6D3) | human, mouse, rat, rabbit, dog, chicken, hamster |  |  | 1:10 | Novocastra/Leica, Wetzlar, Germany |
| dystrophin, C-terminal (Dys2) | mouse IgG1 | monoclonal (Dy8/6C5) |  | 1:100 | 1:10 | 1:10 | Novocastra/Leica, Wetzlar, Germany |
| dystrophin, C-terminal (H4) | rabbit IgG | polyclonal |  | 1:200 |  |  | gift from Cyrille Vailland |
| dystrophin, rod-domain (MANDYS19) | mouse IgG_1_ | monoclonal (86F) | human, mouse | 1:25 | 1:10 |  | DSHB, IO, USA |
| FLAG | rabbit IgG | polyclonal |  |  | 1:200 |  | Sigma-Aldrich, Taufenkirchen, Germany |
| GFAP | mouse IgG1 | monoclonal (G-A-5) | pig, rat, human |  | 1:400 |  | Sigma-Aldrich, Taufenkirchen, Germany |
| GFP | mouse IgG1 | monoclonal (7.1 and 13.1) |  | 1:1,000 | 1:500 |  | Roche, Grenzach-Wyhlen, Germany |
| laminin | rabbit IgG | polyclonal |  |  | 1:500 |  | Sigma-Aldrich, Taufenkirchen, Germany |
| laminin-α2 | rat IgG_1_ | monoclonal (4H8-2) | human, mouse |  |  | 1:100 | Alexis Biochemicals, CA, USA |
| nNOS | rabbit IgG | polyclonal | human, rat, mouse |  |  | 1:1,000 | Upstate/ Millipore, Darmstadt, Germany |
| α-sarcoglycan | mouse IgG_1_ | monoclonal (Ad1/20A6) | human, mouse |  |  | 1:100 | Novocastra/Leica, Wetzlar, Germany |
| β-sarcoglycan | mouse IgG_1_ | monoclonal (β-Sarc/5B1) | human, mouse |  |  | 1:100 | Novocastra/Leica, Wetzlar, Germany |
| γ-sarcoglycan | mouse IgG_2b_, kappa | monoclonal,  (35DAG/21B5) | human, mouse |  |  | 1:100 | Novocastra/Leica, Wetzlar, Germany |
| β-spectrin | mouse IgG_1_ | monoclonal  (RBC1/5B1) | human, rat, mouse, rabbit |  | 1:25 |  | Novocastra/Leica, Wetzlar, Germany |
| utrophin | mouse IgG_1_ | monoclonal  (DRP3/20C5) | human, rat, dog |  | 1:10 |  | Novocastra/Leica, Wetzlar, Germany |
| vinculin | mouse IgG_1_ | monoclonal (SPM227) | mouse, rat, hamster, human | 1:10,000 |  |  | Abcam, UK |

Table S2: Secondary antibodies

| **Antigen** | **Isotype** | **Conjugate** | **Dilutions** | | | **Supplier** |
| --- | --- | --- | --- | --- | --- | --- |
|  |  |  | **WB** | **IF** | **IHC** |  |
| mouse IgG | goat IgG | HRP | 1:2,000 |  | 1:2,000 | Calbiochem/Merck, Darmstadt, Germany |
| mouse IgG_1_ | goat IgG | Alexa-488 |  | 1:400 |  | LifeTechnologies, Darmstadt, Germany |
| mouse IgG1 | goat IgG | Alexa-568 |  | 1:400 |  | LifeTechnologies, Darmstadt, Germany |
| mouse IgG | goat IgG | Alexa-700 | 1:2,000 |  |  | LifeTechnologies, Darmstadt, Germany |
| rabbit IgG | goat IgG | HRP | 1:2,000 |  | 1:2,000 | Calbiochem/Merck, Darmstadt, Germany |
| rabbit IgG | goat IgG | Alexa-568 |  | 1:400 |  | LifeTechnologies, Darmstadt, Germany |
| rabbit IgG | goat IgG | Alexa-800 | 1:2,000 |  |  | LifeTechnologies, Darmstadt, Germany |
